# Lysophosphatidylserines derived from microbiota in Crohn’s disease elicit pathological Th1 response

**DOI:** 10.1084/jem.20211291

**Published:** 2022-05-24

**Authors:** Yuriko Otake-Kasamoto, Hisako Kayama, Toshihiro Kishikawa, Shinichiro Shinzaki, Taku Tashiro, Takahiro Amano, Mizuki Tani, Takeo Yoshihara, Bo Li, Haruka Tani, Li Liu, Akio Hayashi, Daisuke Okuzaki, Daisuke Motooka, Shota Nakamura, Yukinori Okada, Hideki Iijima, Kiyoshi Takeda, Tetsuo Takehara

**Affiliations:** 1 Department of Gastroenterology and Hepatology, Graduate School of Medicine, Osaka University, Osaka, Japan; 2 Laboratory of Immune Regulation, Department of Microbiology and Immunology, Graduate School of Medicine, Osaka University, Osaka, Japan; 3 WPI Immunology Frontier Research Center, Osaka University, Osaka, Japan; 4 Institute for Advanced Co-Creation Studies, Osaka University, Osaka, Japan; 5 Department of Statistical Genetics, Graduate School of Medicine, Osaka University, Osaka, Japan; 6 Department of Otorhinolaryngology—Head and Neck Surgery, Graduate School of Medicine, Osaka University, Osaka, Japan; 7 Discovery Technology Research Laboratories, Ono Pharmaceutical Co., Ltd., Osaka, Japan; 8 Genome Information Research Center, Research Institute for Microbial Diseases, Osaka University, Osaka, Japan; 9 Department of Infection Metagenomics, Research Institute for Microbial Diseases, Osaka University, Osaka, Japan; 10 Integrated Frontier Research for Medical Science Division, Institute for Open and Transdisciplinary Research Initiatives, Osaka University, Suita, Japan

## Abstract

Microbiota alteration and IFN-γ–producing CD4^+^ T cell overactivation are implicated in Crohn’s disease (CD) pathogenesis. However, it remains unclear how dysbiosis enhances Th1 responses, leading to intestinal inflammation. Here, we identified key metabolites derived from dysbiotic microbiota that induce enhanced Th1 responses and exaggerate colitis in mouse models. Patients with CD showed elevated lysophosphatidylserine (LysoPS) concentration in their feces, accompanied by a higher relative abundance of microbiota possessing a gene encoding the phospholipid-hydrolyzing enzyme phospholipase A. LysoPS induced metabolic reprogramming, thereby eliciting aberrant effector responses in both human and mouse IFN-γ–producing CD4^+^ T cells. Administration of LysoPS into two mouse colitis models promoted large intestinal inflammation. LysoPS-induced aggravation of colitis was impaired in mice lacking *P2ry10* and *P2ry10b*, and their CD4^+^ T cells were hyporesponsive to LysoPS. Thus, our findings elaborate on the mechanism by which metabolites elevated in patients with CD harboring dysbiotic microbiota promote Th1-mediated intestinal pathology.

## Teaser

This study shows that Crohn’s disease–associated microbiota generate lysophosphatidylserines that promote Th1 responses by fueling glycolysis. Lysophosphatidylserine-induced aggravation of colitis is prevented by *P2y10* deficiency in CD4^+^ T cells, demonstrating that dysbiotic microbiota-derived LysoPS exacerbates colitis by modulating Th1 cell metabolism.

## Introduction

Crohn’s disease (CD), the main inflammatory bowel disease clinical phenotype, is a chronic gastrointestinal tract disorder with transmural inflammation with unknown etiology. The accumulation of T helper 1 (Th1) cells that produce proinflammatory cytokines, such as IFN-γ and TNF-α, in the intestine is associated with CD severity (Imam et al., 2018; Roda et al., 2020). Along with dysregulated host immune responses, microbial community alterations (referred to as dysbiosis) are a feature of CD (Baumgart and Sandborn, 2012; Levine et al., 2019; Ryan et al., 2020), and microbial network perturbations have been linked to the recurrence of refractory CD (Sokol et al., 2008; Yilmaz et al., 2019). A recent study demonstrated that a strain of *Escherichia coli* enriched in the small intestine of patients with CD promotes accumulation of IFN-γ–producing CD4^+^ T cells in the intestine and induces intestinal inflammation (Nagayama et al., 2020). The human gut microbiome confers benefits to its host through fermentation, nutrient biosynthesis, and host-derived metabolite modification. However, the physiological or pathological functions of many microbial metabolites in the gut remain poorly understood.

We previously reported that some lysophospholipid species were elevated in the plasma of patients with CD (Iwatani et al., 2020). Lysophospholipids are divided into two groups: lysosphingolipids, including sphingosine-1-phosphate (S1P), and lysoglycerophospholipids, comprised of seven lipid classes including lysophosphatidylserine (LysoPS; Tan et al., 2020). The enzyme phospholipase A (PLA) generates lysoglycerophospholipids by hydrolyzing cell membrane phospholipid molecules (Tan et al., 2020). Host-derived PLA exerts antimicrobial activity via bacterial wall penetration (Mukherjee and Hooper, 2015); in contrast, several bacteria produce PLA for colonization during invasion and host cell membrane disruption (Istivan and Coloe, 2006). Our previous lipidomic analysis demonstrated that plasma LysoPS and S1P concentrations were higher in patients with CD than in healthy individuals (Iwatani et al., 2020). The roles of S1P in regulating innate and adaptive immunity are relatively understood (Peyrin-Biroulet et al., 2017). LysoPS, which is generated by host PLA, is shown to regulate several aspects of inflammatory responses. These include suppression of TNF-α and IL-2 production from microglia and splenic CD4^+^ T cells, respectively, inhibition of regulatory T cell (Treg) differentiation, and granulocyte degranulation (Barnes et al., 2015; Makide et al., 2014; Wang et al., 2016). However, much less is known about how LysoPS is generated in the intestine and whether it modulates gut homeostasis.

In this study, to identify the mechanism of action of dysbiosis-dependent lipid metabolites on gut immunity, we performed lipidomic analysis and shotgun metagenomic sequencing (shotgun-seq). We observed that LysoPS was increased in feces of patients with CD, in addition to elevated relative abundance of microbes with the gene encoding phospholipid-hydrolyzing enzyme PLA. LysoPS elicited IFN-γ production in CD4^+^ T cells by fueling glycolysis. Lack of *P2ry10* and *P2ry10b* provided lower responsiveness to LysoPS for CD4^+^ T cells, which led to suppression of LysoPS-dependent aggravation of colitis. Thus, escalation of intestinal LysoPS concentration due to dysbiosis causes progression of pathological Th1 cell–mediated intestinal inflammation.

## Results

### LysoPS was increased in patients with CD

**Figure 1.**
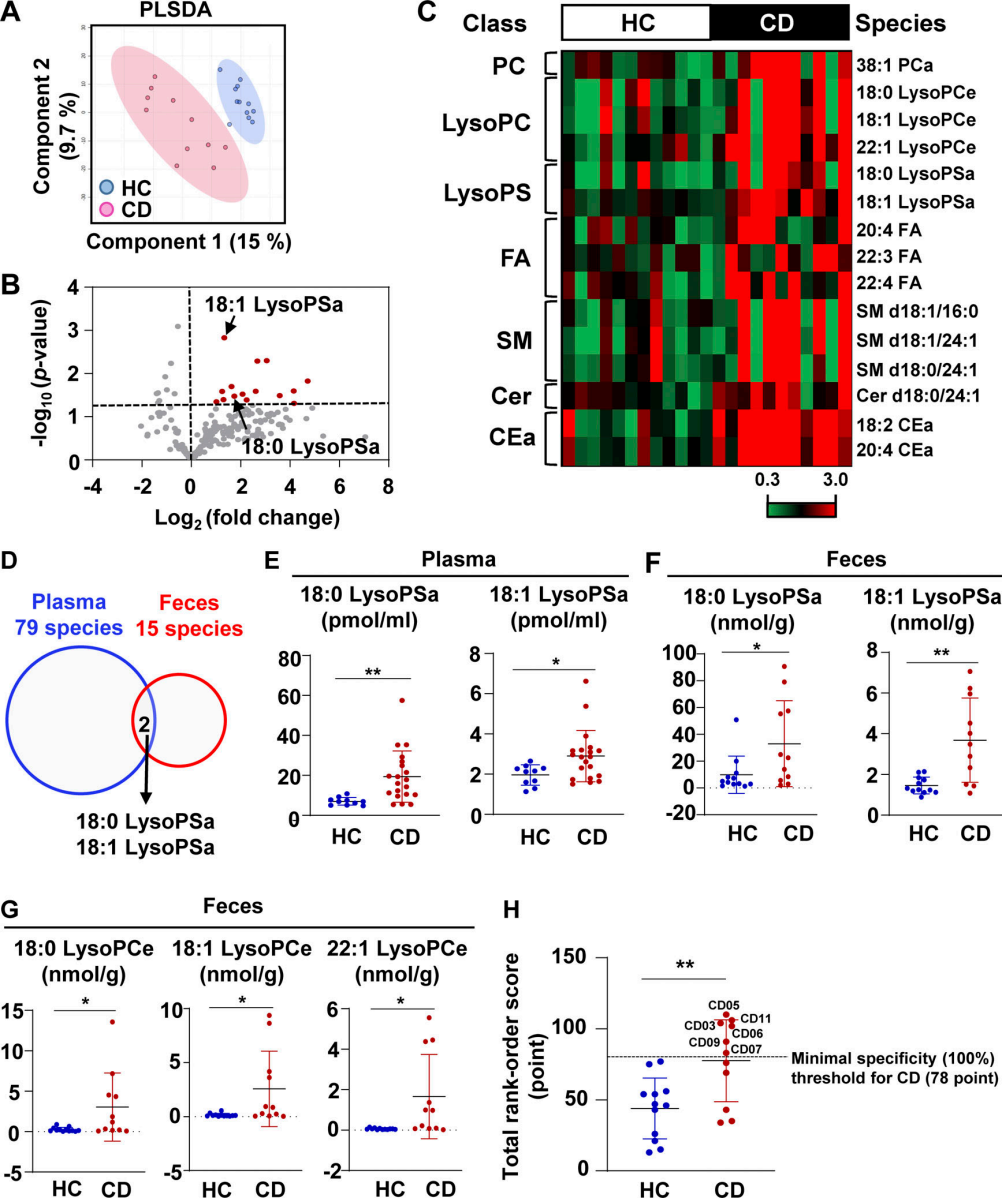
**LysoPS increased in the intestine of CD patients.** A fecal lipidomic analysis was performed on samples from 12 HCs and 11 CD patients. **(A)** Partial least-squares discriminant analysis (PLSDA). **(B)** Volcano plot showing the average log_2_ fold-change for lipidomic data (x axis) and the adjusted negative logarithm base 10 of the P value (y axis). The red circles indicate lipids that were significantly increased in CD samples. P < 0.05; moderated *t* test, adjusted by the Benjamini–Hochberg method. **(C)** Fecal levels of 15 lipid species, presented as fold-change to the average level of each lipid metabolite in HCs. **(D)** Venn diagram showing the overlapping lipid species that were upregulated among the CD plasma (blue) and fecal (red) samples. **(E and F)** Concentrations of 18:0 LysoPS and 18:1 LysoPS in plasma from 10 HCs and 20 patients with CD (E) and feces from 12 HCs and 11 patients with CD (F); *, P < 0.05; **, P < 0.01. **(G)** Concentrations of alkyl ether-linked lysophosphatidylcholine (LysoPCe) species in the fecal samples of 12 HCs and 11 CD patients. *, P < 0.05. **(H)** Rank-order fecal concentration of the lysophospholipids in 12 HCs or 11 patients with CD. Dashed line indicates minimal specificity for the patients (Fig. S1 B). Six patients were above minimal specificity threshold. **, P < 0.01.

**Figure S1.**
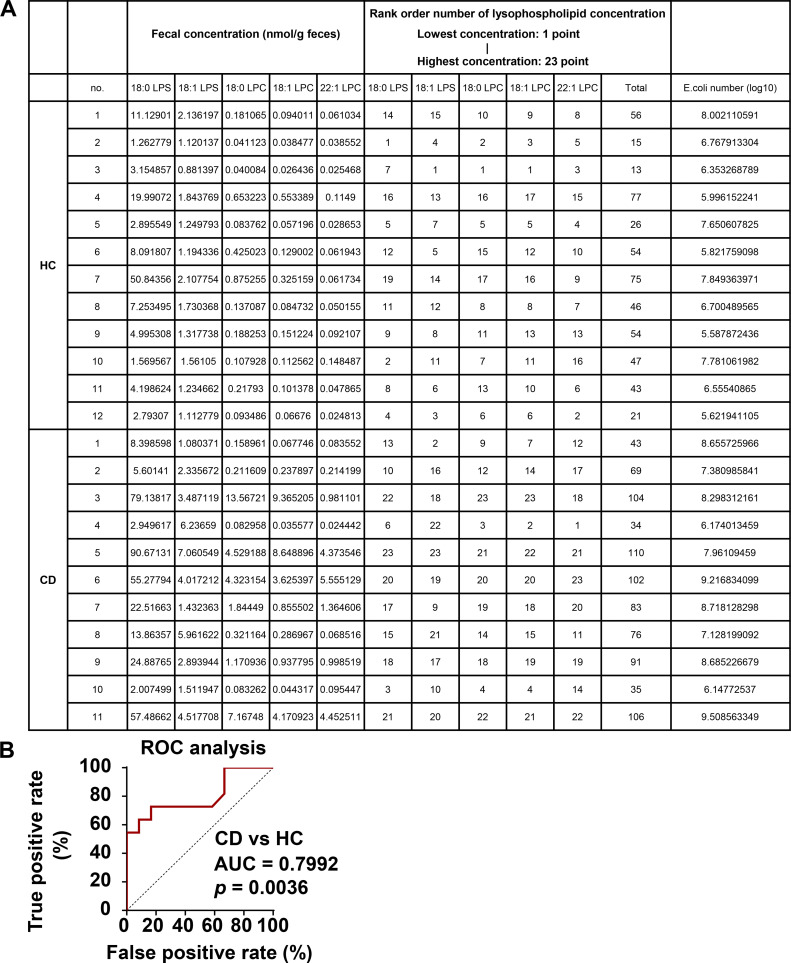
**Elevated concentration of some lysophospholipids in feces derived from patients with CD. (A)** The list of fecal concentration of lysophospholipids, their rank-order score, and the number of *E. coli* in 12 HCs and 11 patients with CD. A rank-order scoring system assigned a normalized value from 1 (lowest) to 23 (highest) for fecal concentration within each lysophospholipid species including 18:0 LysoPS, 18:1 LysoPS, 18:0 LysoPC, 18:1 LysoPC, and 22:1 LysoPC. **(B)** The area under the curve (AUC) in the ROC analysis was 0.7992, which was different from 0.5 (P = 0.0036), indicating that this rank-order scoring could discriminate patients with CD from HCs. HC group, *n* = 12; CD group, *n* = 11.

To define whether plasma lipid profiles, such as LysoPS upregulation in patients with CD (Iwatani et al., 2020), reflect intestinal lipid profiles, we performed a fecal lipidomic analysis. We identified 529 lipid molecular species, belonging to 34 classes, in fecal samples from patients with CD and healthy controls (HCs; Table S1). Partial least-squares discriminant analysis revealed that fecal lipid compositions clearly differed between samples from HCs and patients with CD (Fig. 1 A). 15 lipid species in seven classes were upregulated in fecal samples from patients with CD relative to those from HCs (Fig. 1, B and C; and Table S2). A cross-comparison analysis revealed that augmentation of 18:0 acyl-linked LysoPS (LysoPSa) and 18:1 LysoPSa was common among both the fecal and plasma samples from patients with CD (Fig. 1, D–F). In addition to LysoPS, some lysophosphatidylcholine (LysoPC) species were also found in higher concentrations in the fecal samples of patients with CD than in those of HCs (Fig. 1 G). To more precisely examine whether the upregulation of fecal LysoPS and LysoPC correlates with CD, we introduced a rank-order scoring model (Cao et al., 2017), in which a normalized value from 1 (lowest) to 23 (highest) for fecal concentration within each lysophospholipid species was assigned (Fig. S1 A). Total rank-order score exhibited augmentation of the lysophospholipids in feces in the CD group compared with the HC group (Fig. 1 H). The receiver operating characteristic (ROC) analysis (area under the curve = 0.7992, P = 0.0036: Fig. S1 B) showed that the cutoff value of total rank-order score was 78 points, with a minimal specificity (100%) for patients with CD. In this context, we identified 6 patients with CD among 11 patients (54.5%) as high outliers for fecal levels of lysophospholipids. These findings indicate that concentration of some lysophospholipids in the intestine is elevated in a portion of patients with CD.

### CD-associated microbiota promoted LysoPS generation in the intestine

**Figure 2.**
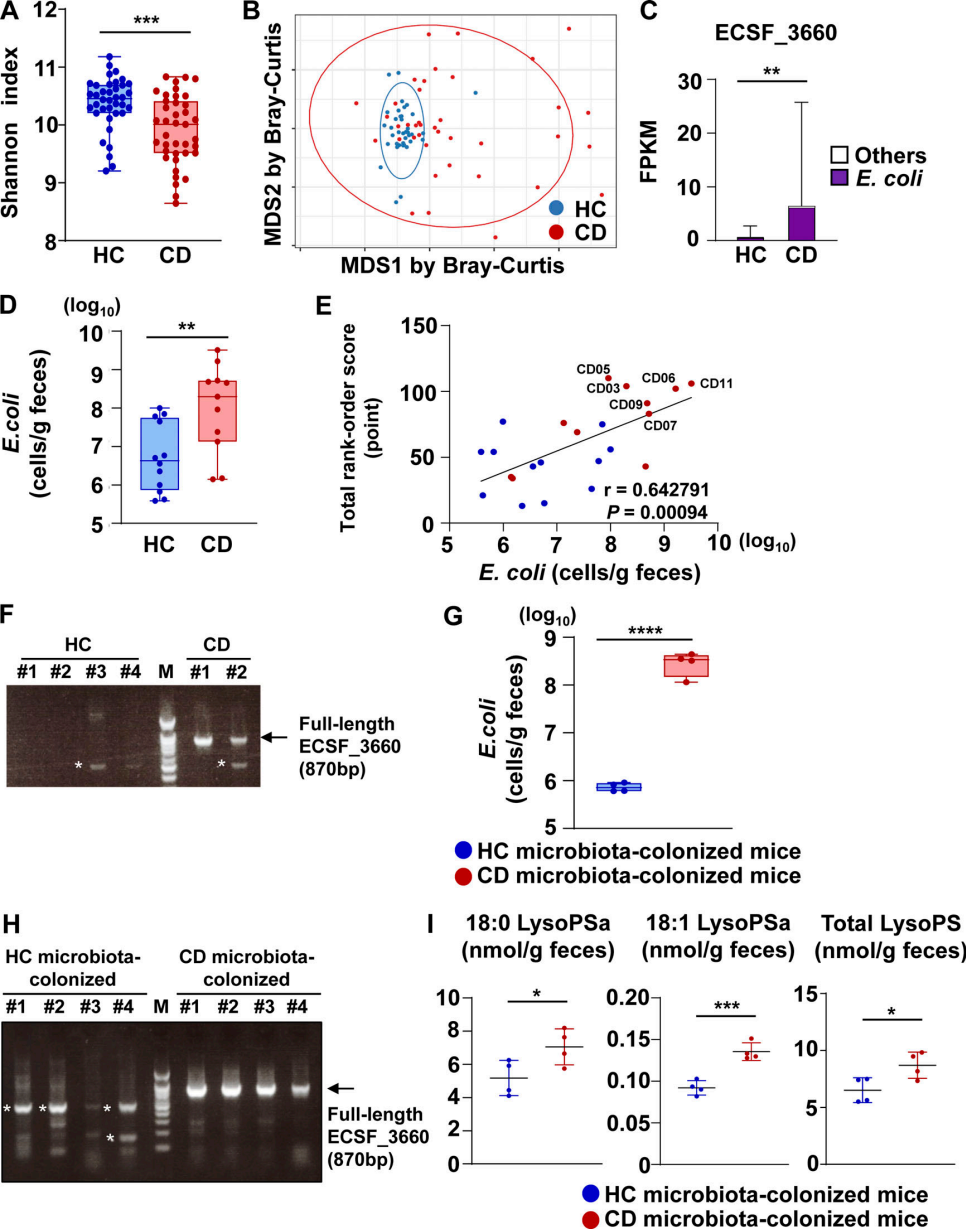
**CD-associated dysbiotic microbiota led to elevated LysoPS production in the intestine. (A–C)** Metagenomic shotgun-seq was conducted on fecal samples from 40 HCs and 43 patients with CD. **(A)** Shannon index for α-diversity. Data were analyzed by Welch’s *t* test. ***, P < 0.005. **(B)** Multidimensional scaling analysis performed using the Bray–Curtis index for β-diversity. Data were analyzed by PERMANOVA. P = 0.0001. **(C)** Fecal abundance of the *ECSF_3660* gene according to the FPKM value (mean values ± SD from 40 HCs or 43 patients with CD). **, P < 0.01. **(D)** The amount of *E. coli* in the fecal samples used for lipidomic analysis. HC group, *n* = 12; CD group, *n* = 11. **, P < 0.01. **(E)** The correlation between the amount of *E. coli* (x axis) and total rank-order score of fecal concentration of lysophospholipids (y axis). HC group, *n* = 12; CD group, *n* = 11. Data were analyzed by simple linear regression. Patient IDs of six outliers (CD03, 05, 06, 07, 09, and 11) identified in Fig. 1 H are represented. **(F)** PCR-based detection of full-length *ECSF_3660* in fecal DNA from four HCs or two CD patients. *, nonspecific band; M, marker. **(G)** The amount of *E. coli* in fecal samples from mice colonized with HC or CD microbiota 10 d after inoculation. *n* = 4/group. ****, P < 0.001. **(H)** PCR-based detection of full-length *ECSF_3660* in fecal DNA from mice colonized with HC or CD microbiota 10 d after inoculation. *n* = 4/group. *, nonspecific band; M, marker. **(I)** Concentration of 18:0, 18:1, and total LysoPS in fecal samples 24 d after microbiota inoculation. *n* = 4/group. *, P < 0.05; ***, P < 0.005. Source data are available for this figure: SourceData F2.

Because PLA in Gram-negative bacteria operates to disrupt the host epithelial cell membrane during invasion (Istivan and Coloe, 2006), we hypothesized that microbiota community alterations in patients with CD led to the generation of more intestinal lysoglycerophospholipids by microbial PLA. To investigate this possibility, we performed whole-genome shotgun-seq of fecal DNA. Lower fecal microbiota species diversity (Shannon index) was observed in CD samples compared with HC samples (Fig. 2 A). Additionally, a β-diversity (Bray–Curtis dissimilarity) analysis demonstrated that the microbial community profiles of CD samples substantially separated from those of HC samples (Fig. 2 B). We then investigated the fecal relative abundances of microbial PLA-encoding genes. Among the seven genes predicted to encode PLA, *ECSF_3660* (mainly derived from *Escherichia coli*) was elevated in the fecal samples of patients with CD compared with HCs (Fig. 2 C). Indeed, the amount of *E. coli* was higher in CD fecal samples (Fig. 2 D). Furthermore, the amount of *E. coli* in feces correlated positively with a relative abundance of fecal lysophospholipids normalized by the rank-order scoring (Fig. 2 E); the six outliers identified in Fig. 1 H harbored high numbers of *E. coli*. Therefore, to examine whether dysbiotic microbiota causes elevated LysoPS concentrations in the intestines of patients with CD, germ-free mice were inoculated with a mixture of feces from two patients with CD exhibiting detectable amounts of *ECSF_3660* or from four HCs (Fig. 2 F). Gnotobiotic mice colonized with microbiota from patients with CD showed an increased amount of *E. coli* in their feces relative to mice transplanted with HC-derived microbiota 10 d after inoculation (Fig. 2 G). Additionally, we detected *ECSF_3660* among microbial DNA from the feces of mice harboring microbiota from patients with CD, but not in those transplanted with healthy microbiota (Fig. 2 H). We further measured the fecal concentrations of 121 lipid species 24 d after inoculation (Table S3). Among them, 30 lipid species in the seven classes were higher in the feces from mice harboring microbiota from patients with CD (Table S4). Fecal concentrations of 18:0 LysoPSa, 18:1 LysoPSa, and total LysoPSa were elevated in mice transplanted with the microbial community of patients with CD compared with those in mice harboring healthy microbiota (Fig. 2 I). These findings suggest that colonization by the dysbiotic microbiota found in patients with CD links to the promotion of LysoPS generation in the intestine.

### LysoPS exacerbated T cell–mediated colitis by promoting IFN-γ–producing CD4^+^ T cell accumulation in the colon

**Figure S2.**
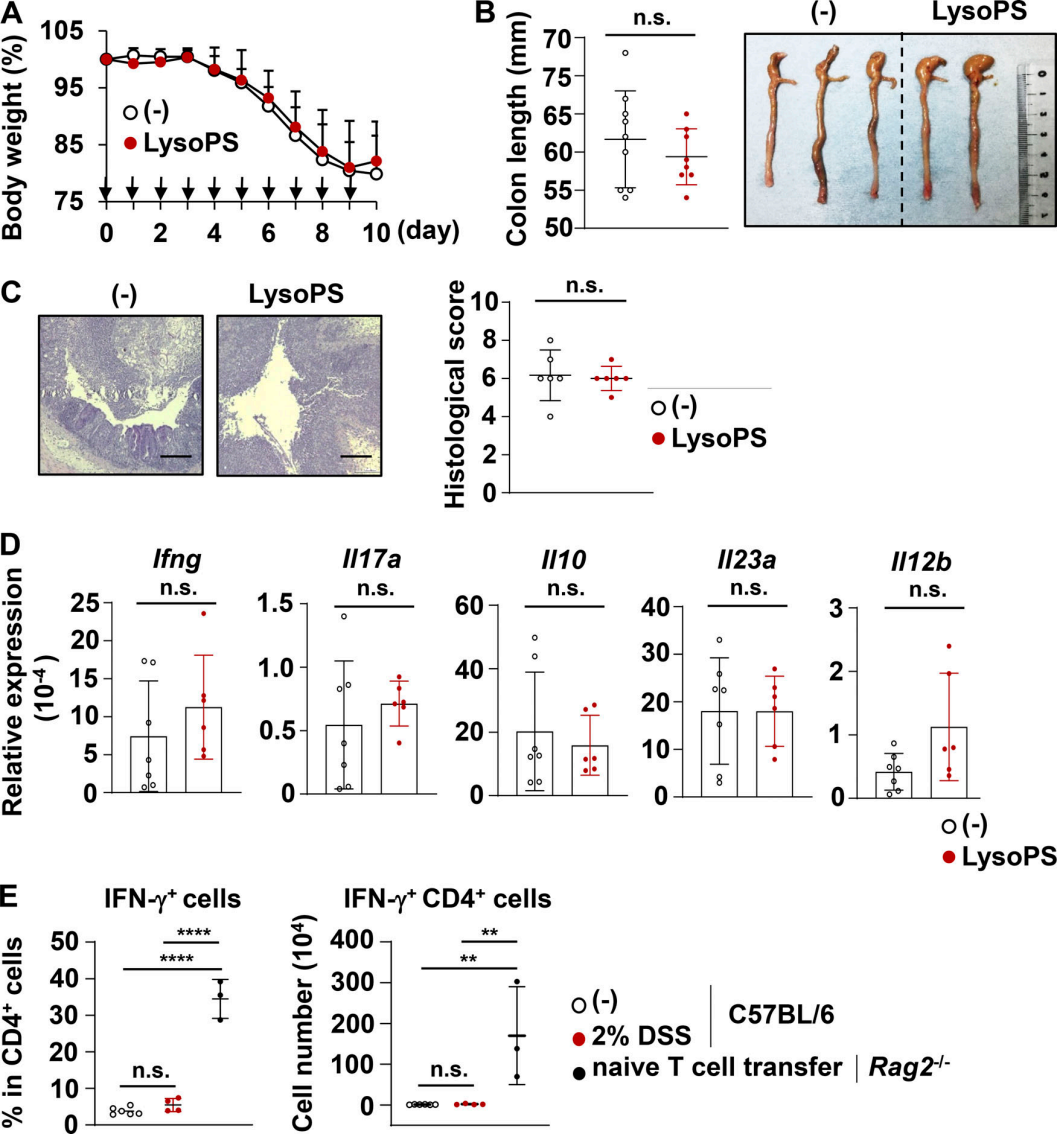
**18:1 LysoPS did not affect DSS-induced colitis.** C57BL/6J mice were administered 2% DSS and intraperitoneally injected with 18:1 LysoPS or vehicle once daily for 10 d. **(A)** Body weight changes of mice treated with LysoPS (*n* = 8) or vehicle (*n* = 9; mean values ± SD). Arrows indicate LysoPS injection time points. Data were pooled from four independent experiments. **(B)** The colon lengths of LysoPS-injected (*n* = 8) or vehicle-injected (*n* = 9) mice (mean values ± SD). Data were pooled from four independent experiments. **(C)** Left: Representative distal colon sections. Right: Histological score. Data show mean ± SD from six mice/group. Data were pooled from three independent experiments. **(D)** Expression levels of the indicated genes in colonic lamina propria cells from LysoPS- or vehicle-injected mice. Graphs present mean ± SD of 6–7 mice. All data were pooled from three independent experiments. **(E)** C57BL/6J mice were treated with (*n* = 4) or without (*n* = 6) 2% DSS. The large intestinal tissues of these mice were isolated 8 d after initiation of DSS administration, and percentage (left) and the number (right) of IFN-γ^+^ CD4^+^ T cells were analyzed. *Rag2*^−/−^ mice received transferred naive CD4^+^ T cells (5 × 10^5^) magnetically isolated from the spleens of C57BL/6J mice (*n* = 3); 21 d later, the percentage (left) and the number (right) of IFN-γ^+^ CD4^+^ T cells in the colon were analyzed in these mice. **, P < 0.01; ****, P < 0.001. Scale bars: 200 μm.

To determine the role of LysoPS generated by dysbiotic microbiota in the progression of CD, 18:1 LysoPS was administered to several mouse colitis models. We first investigated the effect of 18:1 LysoPS on the pathogenesis of dextran sodium sulfate (DSS)–induced colitis. LysoPS did not affect body weight loss during DSS administration (Fig. S2 A). In accordance with these results, there were no differences in colon length, colon histopathology, or the gene expression levels of *Ifng*, *Il17a*, *Il10*, *Il12b*, and *Il23a* in lamina propria cells between LysoPS-treated and untreated mice (Fig. S2, B–D).

**Figure 3.**
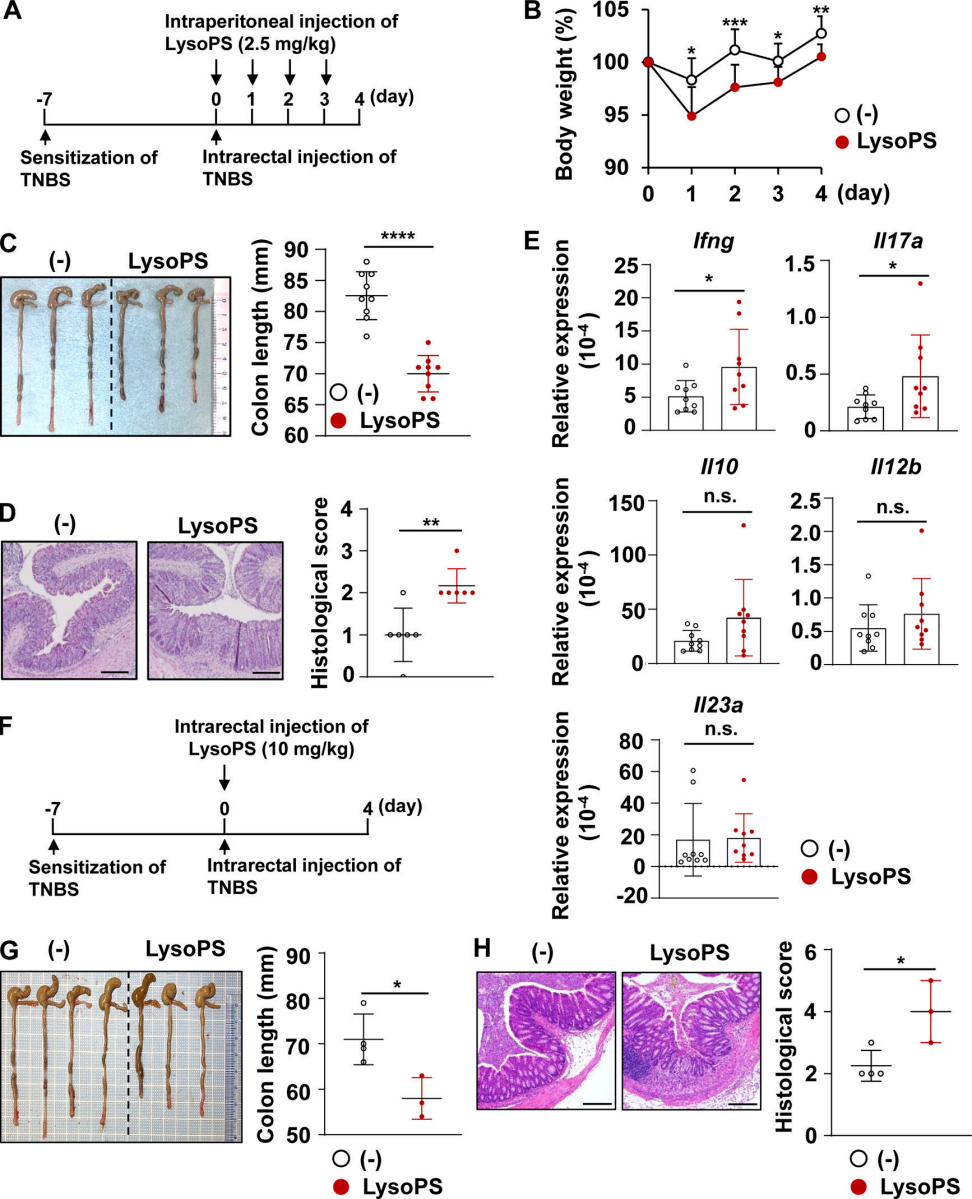
**LysoPS exacerbated TNBS-induced colitis. (A)** The scheme of the experiments. **(B)** Body weight changes of control and LysoPS-treated mice (*n* = 9/group; mean values ± SD). *, P < 0.05; **, P < 0.01; ***, P < 0.005. All data were pooled from three independent experiments. Arrows indicate LysoPS injection time points. **(C)** Left: Representative colon images. Right: Colon length. Graphs show mean ± SD from nine mice/group pooled from three independent experiments. ****, P < 0.001. **(D)** Left: Representative distal colon sections. Right: Histological scores. Mean values ± SD from six mice/group. Data were pooled from three independent experiments. **, P < 0.01. **(E)** Expression levels of the indicated genes in the colon from nine mice/group. Data were pooled from three independent experiments (mean ± SD). *, P < 0.05. **(F)** The scheme of the experiments. **(G)** Left: Representative colon images. Right: Colon length. Graphs show mean ± SD from four (vehicle) or three (LysoPS) mice from one experiment. *, P < 0.05. **(H)** Left: Representative distal colon sections. Right: Histological scores. Mean values ± SD from four (vehicle) or three (LysoPS) mice/group from one experiment. *, P < 0.05. Scale bars: 200 μm.

We next analyzed the effect of 18:1 LysoPS on 2,4,6-trinitrobenzenesulfonic acid solution (TNBS)–induced colitis in C57BL/6 mice. Mice sensitized with TNBS were intrarectally administered TNBS with or without an intraperitoneal injection of 18:1 LysoPS (Fig. 3 A). Mice with LysoPS treatment exhibited severe body weight loss and colon shortening (Fig. 3, B and C); these were associated with worse colonic histopathology (Fig. 3 D). Those mice were analyzed for mRNA expression of cytokines, such as *Ifng*, *Il17a*, *Il10*, *Il12b*, and *Il23a* in the lamina propria cells (Fig. 3 E). Expression of *Ifng* and *Il17a* was higher in LysoPS-treated mice than untreated mice, whereas expression of *Il10*, *Il12b*, and *Il23a* was not changed. Moreover, mice administered 18:1 LysoPS intrarectally suffered from more severe TNBS-induced colitis, as evidenced by shortening of the colon and increased histopathological scores compared with those in control mice (Fig. 3, F–H).

**Figure 4.**
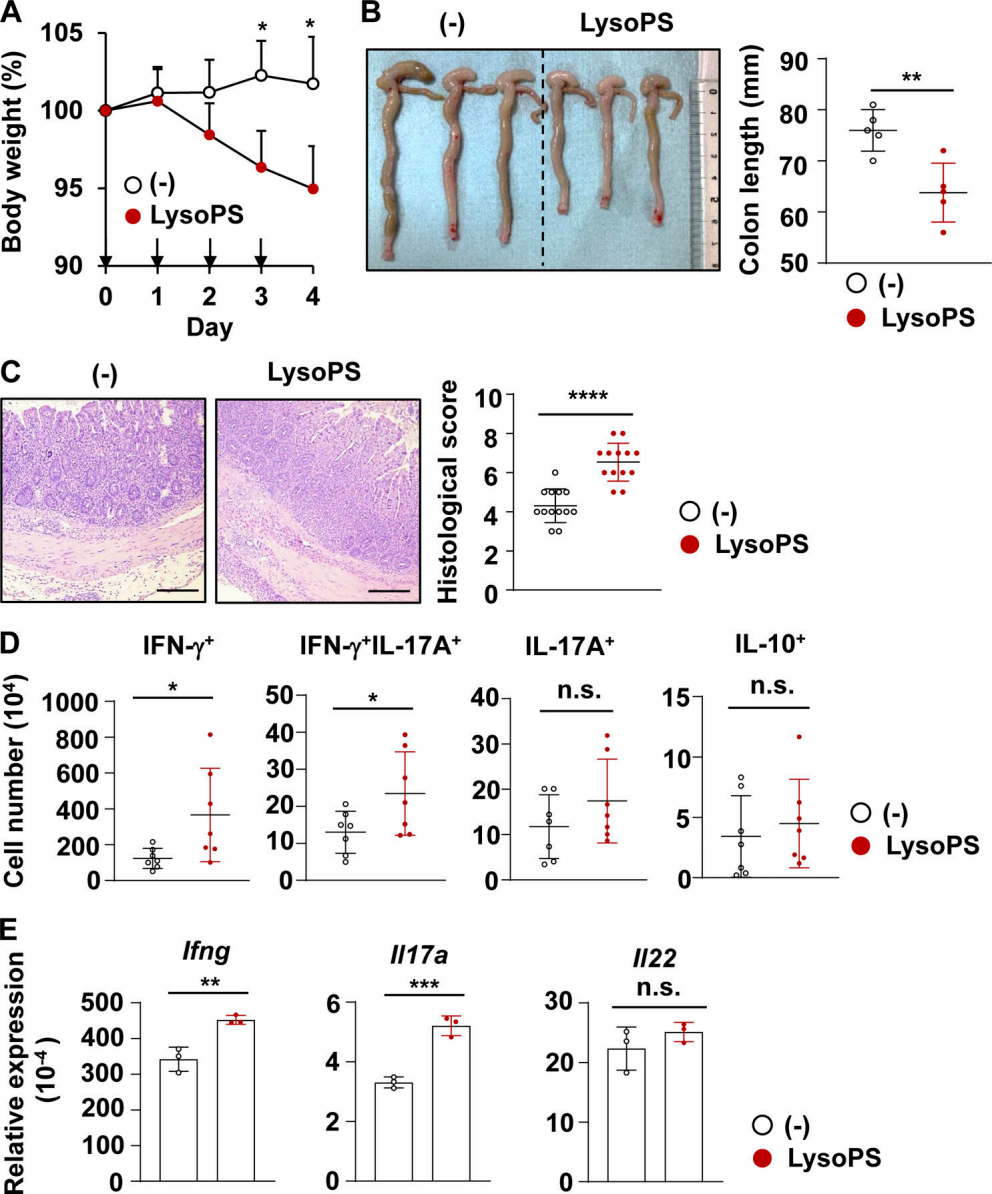
**LysoPS exacerbated T cell–dependent intestinal inflammation. (A)** Body weight changes of mice injected with LysoPS (or vehicle, indicated as −; *n* = 4/group; mean ± SD). Data are representative of three independent experiments. *, P < 0.05. **(B)** The colon length. Data were pooled from three independent experiments. Mean ± SD from five mice/group is shown. **, P < 0.01. **(C)** Left: Representative distal colon sections. Right: Histological scores (mean ± SD from group of 13 mice). Data are from five independent experiments. ****, P < 0.001. **(D)** The numbers of indicated cells from colonic lamina propria. Data were pooled from three independent experiments and are presented as mean values ± SD from seven mice/group. *, P < 0.05. **(E)** Expression level of the indicated genes in colonic lamina propria cells from naive T cell–transferred *Rag2*^−/−^ mice that were injected or not with LysoPS (mean ± SD). *n* = 3/group from one experiment. **, P < 0.01; ***, P < 0.005. Scale bars: 200 μm.

We hypothesized that the contrasting results between these two colitis models were caused by the differential dependence on T cell responses (Wirtz et al., 2007). To confirm it, we next analyzed the effect of LysoPS on an adoptive T cell transfer-induced colitis model. Naive CD4^+^ T cells were transferred into *recombination activating gene 2*–deficient (*Rag2*^−/−^) mice. Beginning 17 d later, these mice received an intraperitoneal injection of 18:1 LysoPS. Compared to untreated *Rag2*^−/−^ mice that received naive CD4^+^ T cells, profound body weight loss accompanied by severe colon length shortening was observed in LysoPS-treated *Rag2*^−/−^ mice (Fig. 4, A and B). Moreover, histological analysis revealed LysoPS-induced aggravation of large intestinal pathology (Fig. 4 C). In accordance with the higher colitis severity of this model, the numbers of IFN-γ^+^ and IFN-γ^+^ IL-17A^+^ CD4^+^ cells, but not IL-17^+^ or IL-10^+^ CD4^+^ T cells, in the large intestinal lamina propria were higher in LysoPS-treated mice than in control mice (Fig. 4 D). In addition, mRNA expression of *Ifng* and *Il17a*, but not *Il22*, was increased in colonic lamina propria cells of LysoPS-treated mice relative to control mice (Fig. 4 E). In accordance with the contrasting effect of LysoPS on these colitis models, the number of IFN-γ–producing CD4^+^ T cells in the large intestine was substantially increased in *Rag2*^−/−^ mice that received naive CD4^+^ T cells compared with DSS-treated mice (Fig. S2 E). Thus, LysoPS exacerbates colitis by enhancing immunopathological Th1 responses in the intestinal lamina propria.

### LysoPS enhanced Th1 cell effector functions

**Figure 5.**
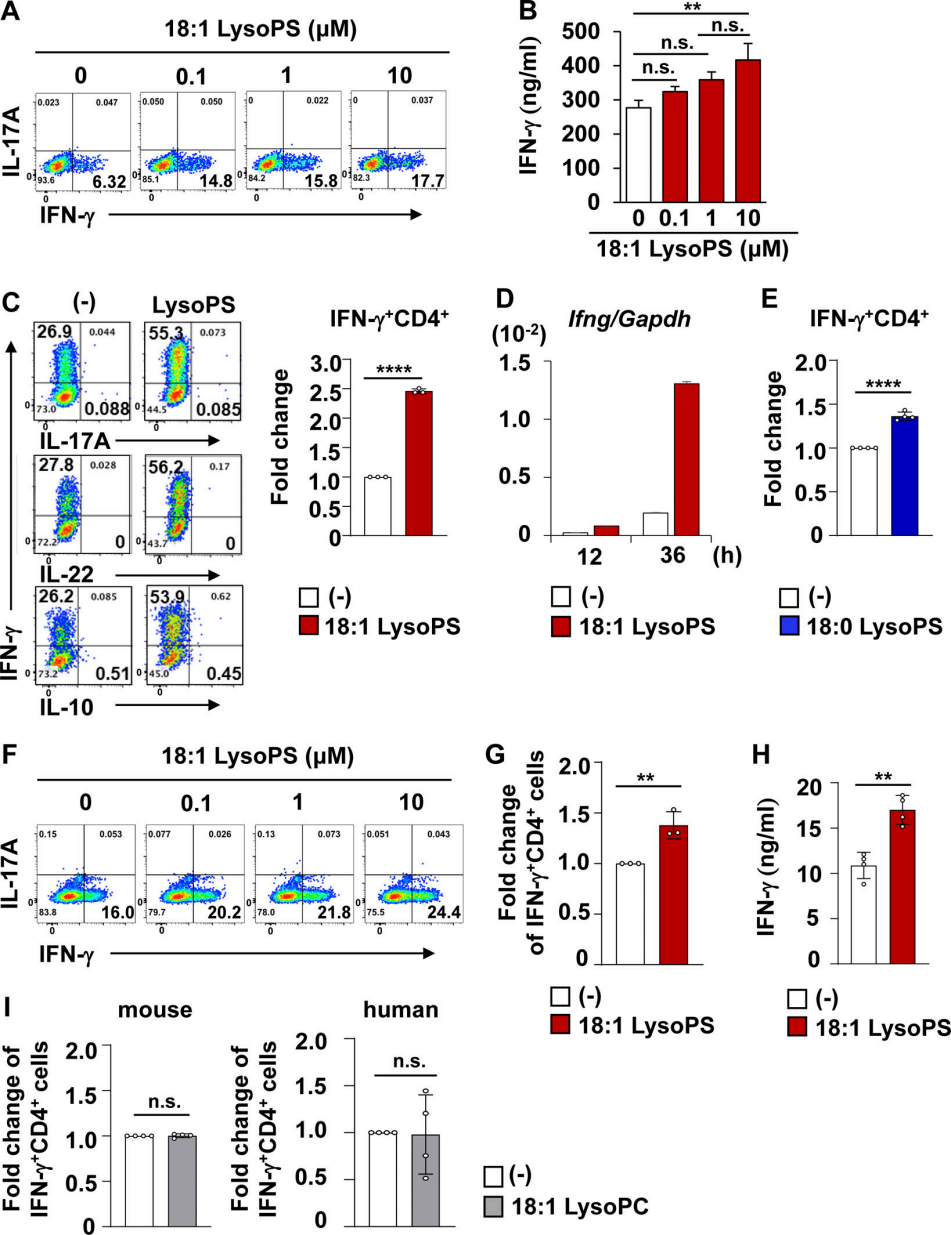
**LysoPS accelerated IFN-γ production in murine and human CD4**^**+**^ **T cells. (A)** Representative flow cytometric plots of IL-17A– and IFN-γ–producing CD4^+^ T cells. **(B)** The amount of IFN-γ in supernatants of splenic mononuclear cells stimulated with 18:1 LysoPS (mean ± SD). Data were pooled from three independent experiments. **, P < 0.01. Data were evaluated by one-way ANOVA. **(C)** Left: Representative flow cytometric plots of IL-17A–, IL-22–, IL-10–, and IFN-γ–producing CD4^+^ T cells from three independent experiments. Right: Fold-change of IFN-γ–producing CD4^+^ cells (mean ± SD from three independent experiments). ****, P < 0.001. **(D)** Expression levels of *Ifng* in CD4^+^ T cells at the indicated time points after LysoPS stimulation. Data are representative of two independent experiments. **(E)** Fold-change of IFN-γ–producing CD4^+^ cells (mean ± SD from four independent experiments). Data show mean ± SD of at least three independent experiments. ****, P < 0.001. **(F)** Representative flow cytometric plots of human IL-17A– and IFN-γ–producing CD4^+^ T cells. **(G)** Fold-change of human IFN-γ–producing CD4^+^ cells (mean ± SD from three independent experiments). **, P < 0.01. **(H)** Concentration of IFN-γ in the culture supernatants (mean ± SD from four independent experiments). **, P < 0.01. **(I)** Fold-change of murine (left) or human (right) IFN-γ–producing CD4^+^ cells (mean ± SD from four independent experiments). Fold-change is presented relative to the percentage of vehicle-treated cells. Vehicle indicated as (−) in all data.

To explore how LysoPS modulates Th1 responses, murine splenic naive CD4^+^ T cells were skewed to Th1 cells in vitro in the presence or absence of 18:1 LysoPS. The percentage of IFN-γ–producing CD4^+^ T cells was increased in a dose-dependent manner by 18:1 LysoPS (Fig. 5 A). Concentration of IFN-γ in the supernatants of LysoPS-stimulated splenocytes was also increased (Fig. 5 B). In Th1-polarized CD4^+^ T cells stimulated with 10 μM LysoPS, the percentage of IFN-γ–producing cells was ∼2.5-fold higher than in untreated cells, but the percentages of IL-17A–, IL-22–, and IL-10–producing cells were not changed (Fig. 5 C). We also observed LysoPS-mediated upregulation of *Ifng* transcription in CD4^*+*^ T cells (Fig. 5 D). Additionally, 18:0 LysoPS increased the percentage of IFN-γ–producing cells in Th1 culture (Fig. 5 E). To investigate whether LysoPS influences human Th1 responses, human blood naive CD4^+^ T cells were cultured under Th1 polarizing conditions. 18:1 LysoPS increased the percentage of IFN-γ–producing CD4^+^ T cells (Fig. 5, F and G). Concentration of IFN-γ in the supernatants of LysoPS-treated human Th1 cells was also elevated (Fig. 5 H). In contrast to LysoPS, 18:1 LysoPC, which was significantly elevated in CD fecal samples, did not drive IFN-γ production in murine and human Th1-polarized CD4^+^ T cells (Fig. 5 I). These findings indicate that LysoPS, but not LysoPC, directly activates IFN-γ production in both murine and human Th1-skewed cells.

### LysoPS modulated bioenergetic metabolism of Th1 cells

**Figure 6.**
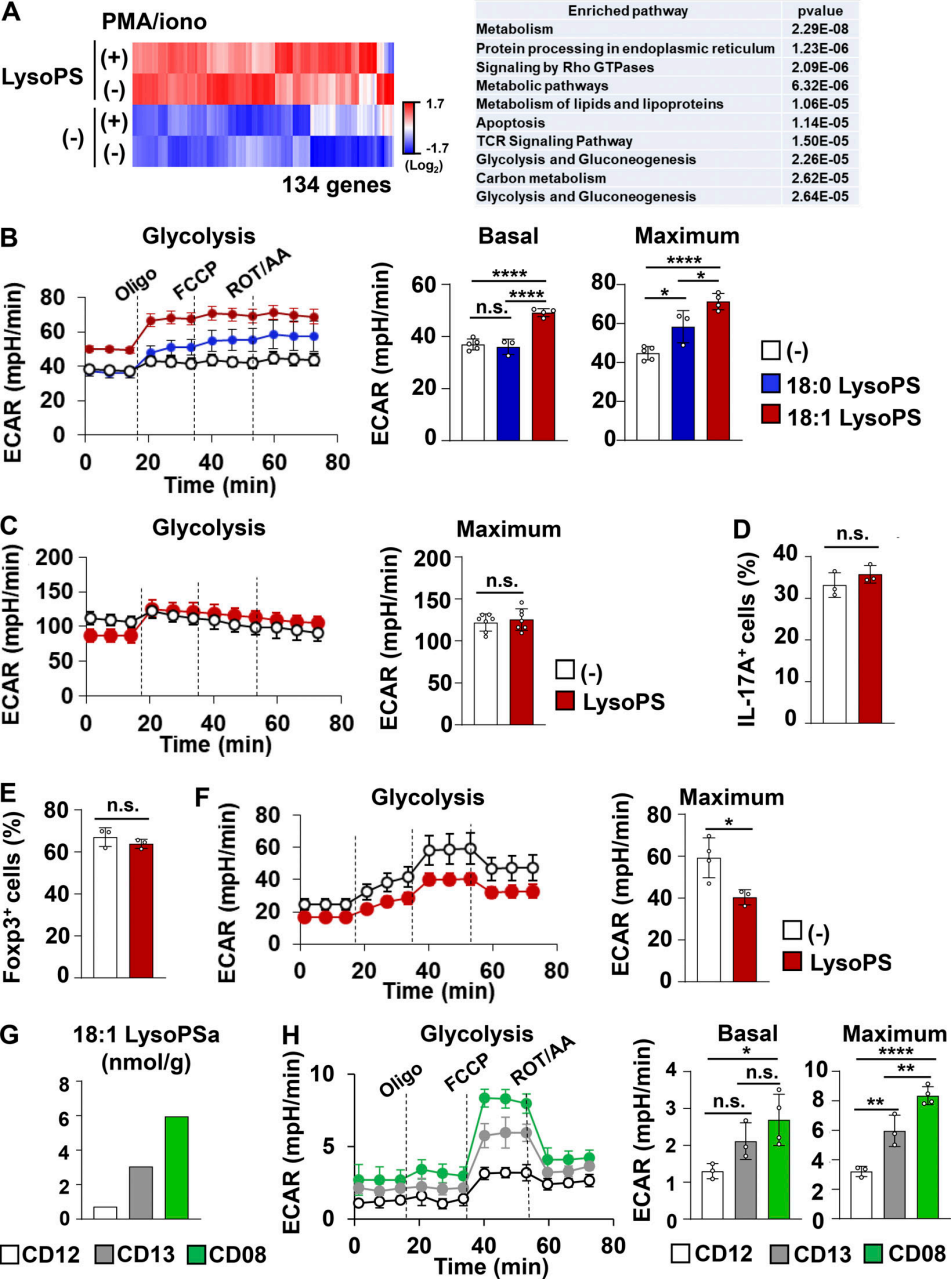
**LysoPS facilitated IFN-γ production by modulating Th1 cell metabolism. (A)** Left: Heatmap of differentially expressed genes. Right: Enriched pathways among the genes in LysoPS-stimulated Th1 cells. **(B)** Basal and maximum ECAR values from at least three wells (mean values ± SD). All data are representative of three independent experiments. *, P < 0.05; ****, P < 0.001. ROT/AA, rotenone/antimycinA. **(C and F)** Maximum ECAR in murine CD4^+^ T cells cultured under Th17 (C) or Treg (F) polarizing conditions (mean values ± SD from at least three wells). *, P < 0.05. **(D and E)** The percentages of IL-17A–producing (D) or Foxp3-expressing (E) CD4^+^ cells (mean ± SD). **(G)** Concentrations of fecal 18:1 LysoPS from three CD patients. **(H)** Basal and maximum ECAR (mean ± SD from at least three wells) from three CD patients. Statistical analysis was performed via one-way ANOVA. *, P < 0.05; **, P < 0.01; ****, P < 0.001.

**Figure S3.**
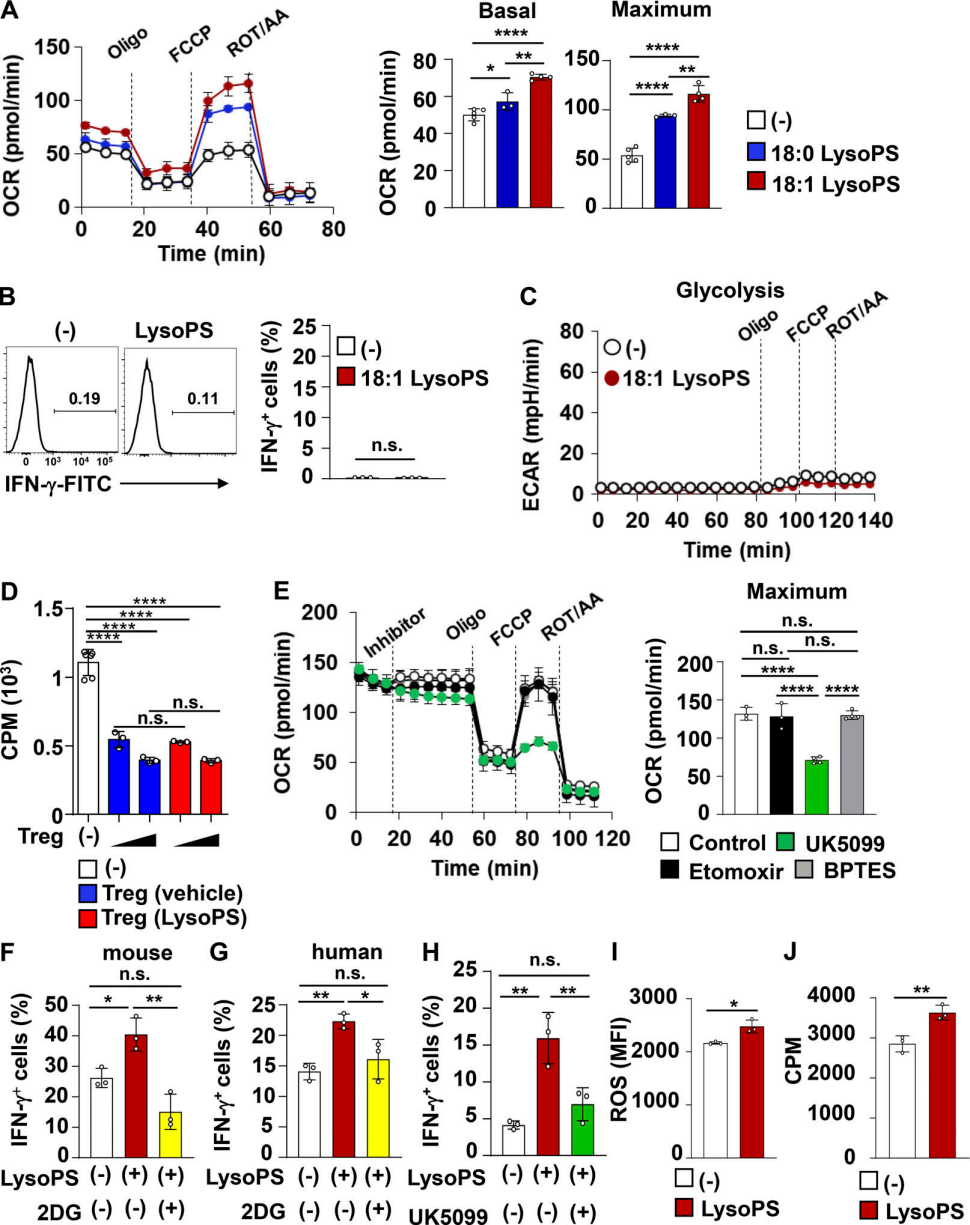
**LysoPS induced metabolic changes in IFN-γ–producing CD4**^**+**^ **T cells. (A)** Basal and maximum OCR in murine CD4^+^ T cells cultured under Th1-polarized conditions with or without LysoPS. Bar graphs show average basal and maximum OCR values from at least three wells (mean values ± SD). All data are representative of three independent experiments. *, P < 0.05; **, P < 0.01; ****, P < 0.001. ROT/AA, rotenone/antimycinA. **(B and C)** Murine naive CD4^+^ T cells from the spleen were cultured under Th0-inducing conditions for 24 h, after which 10 μM 18:1 LysoPS or vehicle (indicated as [−]) was added into the culture for 48 h. **(B)** Left: IFN-γ expression in CD4^+^ T cells. Right: Graphs of the percentage of IFN-γ–producing CD4^+^ cells (mean ± SD from three independent experiments). **(C)** Measurements of ECAR. Data are representative of three independent experiments. **(D)** 0.5 × 10^5^ or 2 × 10^5^ of in vitro–generated murine Treg cells in the presence (red bar) or absence (blue bar) of 10 μM LysoPS were added to co-culture of 1 × 10^5^ of naive CD4^+^ T cells with 1 × 10^4^ of CD11c^+^ splenic dendritic cells; 54 h later, the cells were pulsed with 1 μCi [^3^H]-thymidine for 18 h. ****, P < 0.001. All data were analyzed by one-way ANOVA. ROT/AA, rotenone/antimycinA. **(E)** OCR of CD4^+^ T cells in Th1 culture with or without LysoPS were measured in the presence of the indicated inhibitors. Bar graphs show the average values of maximum OCR from at least three wells (mean values ± SD). n.s., not significant; ****, P < 0.001. **(F and G)** Naive T cells from murine spleens (F) or human blood (G) were cultured under Th1-polarized conditions with or without 2DG and LysoPS. The percentages of IFN-γ–producing CD4^+^ T cells in these populations were measured. All data are from three independent experiments (mean ± SD). *, P < 0.05; **, P < 0.01. All data were analyzed by one-way ANOVA. **(H)** The percentages of IFN-γ–producing CD4^+^ cells cultured under Th1-polarized conditions with or without UK5099 and LysoPS. Graphs show the mean ± SD from three independent experiments; **, P < 0.01. All data were analyzed by one-way ANOVA. **(I)** Intracellular ROS production in CD4^+^ T cells cocultured under Th1-polarized conditions with 10 μM 18:1 LysoPS or vehicle (mean values ± SD from three wells). Data are representative of three independent experiments. *, P < 0.05. **(J)** [^3^H]thymidine uptake by CD4^+^ T cells cocultured under Th1-polarized conditions with 10 μM 18:1 LysoPS or vehicle (mean values ± SD from four wells). **, P < 0.01.

To investigate how LysoPS manipulates T cell effector functions, we analyzed gene expression profiles from in vitro–generated Th1 cells. RNA sequencing (RNA-seq) analysis revealed that LysoPS augments a subset of genes in Th1-polarized cells (Fig. 6 A). Moreover, pathway enrichment analysis within the BioJupies platform (https://amp.pharm.mssm.edu/biojupies/upload/table) identified enriched pathways of the genes upregulated in response to LysoPS. Among the top 10 enriched pathways, 6 were associated with metabolic processes (Fig. 6 A). These findings raise the possibility that LysoPS induces a metabolic change that promotes Th1 cell effector responses. Therefore, we evaluated CD4^+^ T cell bioenergetic profiles by quantifying the extracellular acidification rate (ECAR) and the oxygen consumption rate (OCR). In murine Th1-skewed cells, 18:1 LysoPS markedly upregulated the basal and maximum ECAR and OCR (Figs. 6 B and S3 A), and 18:0 LysoPS promoted the maximum ECAR and the basal and maximum OCR. As 18:1 LysoPS showed potent bioenergetic activity in Th1-polarized cells relative to 18:0 LysoPS, we used 18:1 LysoPS in all following in vitro and in vivo experiments. Unlike Th1-skewed cells, naive CD4^+^ T cells cultured under Th0 conditions were unresponsive to LysoPS, as evidenced by the lack of an elevation of basal and maximum ECAR or a promotion of IFN-γ production during culture in the presence of LysoPS (Fig. S3, B and C). Similarly, Th17-skewed CD4^+^ T cells did not exhibit LysoPS-mediated alteration of maximum ECAR and IL-17A production (Fig. 6, C and D). We then analyzed the effect of LysoPS on Treg cells. LysoPS did not influence in vitro differentiation of naive CD4^+^ T cells into Foxp3^+^ CD4^+^ T cells (Fig. 6 E). However, LysoPS downregulated maximum ECAR in Treg cells (Fig. 6 F). Because metabolic status was affected in LysoPS-treated Treg cells, we analyzed whether LysoPS influences the suppressive function of Treg cells. Treg-polarized cells with or without LysoPS were added to co-culture of naive CD4^+^ T cells and CD11c^+^ dendritic cells (Fig. S3 D). LysoPS-treated Treg cells inhibited T cell proliferation in a similar manner to that observed in vehicle-treated Treg cells, implying that LysoPS-mediated modulation of glycolysis does not militate against the suppressive activity of Treg cells. Thus, these findings indicate that LysoPS promotes Th1 responses through acceleration of glycolysis. During metabolic assay using murine Th1-polarized cells, we added either the glutamine oxidation inhibitor BPTES, fatty acid oxidation inhibitor Etomoxir, or pyruvate carrier inhibitor UK5099. UK5099, but not BPTES or Etomoxir, markedly reduced the maximum OCR in LysoPS-stimulated murine Th1-polarized cells (Fig. S3 E), suggesting that LysoPS might promote mitochondria respiration by providing increased amounts of pyruvate through activation of glycolytic metabolism.

Because LysoPS-mediated promotion of glycolytic activity was shown in murine Th1-skewed cells, we next analyzed whether the increased amounts of LysoPS contributes to the modulation of T cell metabolism in patients with CD. Blood CD4^+^ CD25^−^ CD45RO^+^ effector memory T cells (Cao et al., 2017) were isolated from the patients with high, middle, or low concentrations of fecal LysoPS and measured ECAR (Fig. 6, G and H). Basal and maximum levels of ECAR positively correlated with fecal LysoPS concentration, suggesting that the increased level of LysoPS contributes to the metabolic changes in effector CD4^+^ T cell in patients with CD.

Accumulating evidence demonstrates that glycolysis is an essential metabolic pathway for evoking T cell effector responses (Chang et al., 2013; Macintyre et al., 2014; Peng et al., 2016; Vacanti et al., 2014). Therefore, to investigate whether LysoPS modulates Th1 responses via promoting glycolysis, the glucose analog 2-deoxy-D-glucose (2DG) was added to Th1 differentiation culture. The LysoPS-induced increase in the IFN-γ–producing CD4^+^ T cell percentage was abrogated in both murine and human CD4^+^ T cells by 2DG-dependent glycolysis inhibition (Fig. S3, F and G). Additionally, UK5099 suppressed the LysoPS-mediated induction of IFN-γ–producing CD4^+^ T cells (Fig. S3 H), which indicates that transport of glycolysis-derived pyruvate in mitochondria mediates the LysoPS-dependent acceleration of Th1 responses. In mitochondria, pyruvate is converted to acetyl-CoA, which is required for the TCA cycle and subsequent oxidative phosphorylation (OXPHOS). Production of ROS, a byproduct of OXPHOS, was elevated in Th1-polarized cells in response to LysoPS (Fig. S3 I). LysoPS-mediated promotion of proliferative responses was also observed in in vitro–generated Th1 cells in the presence of LysoPS, as determined by [^3^H]thymidine incorporation (Fig. S3 J). These findings demonstrate that LysoPS facilitates Th1 responses by enhancing glycolysis.

### LysoPS induced epigenetic changes in Th1 cells

**Figure 7.**
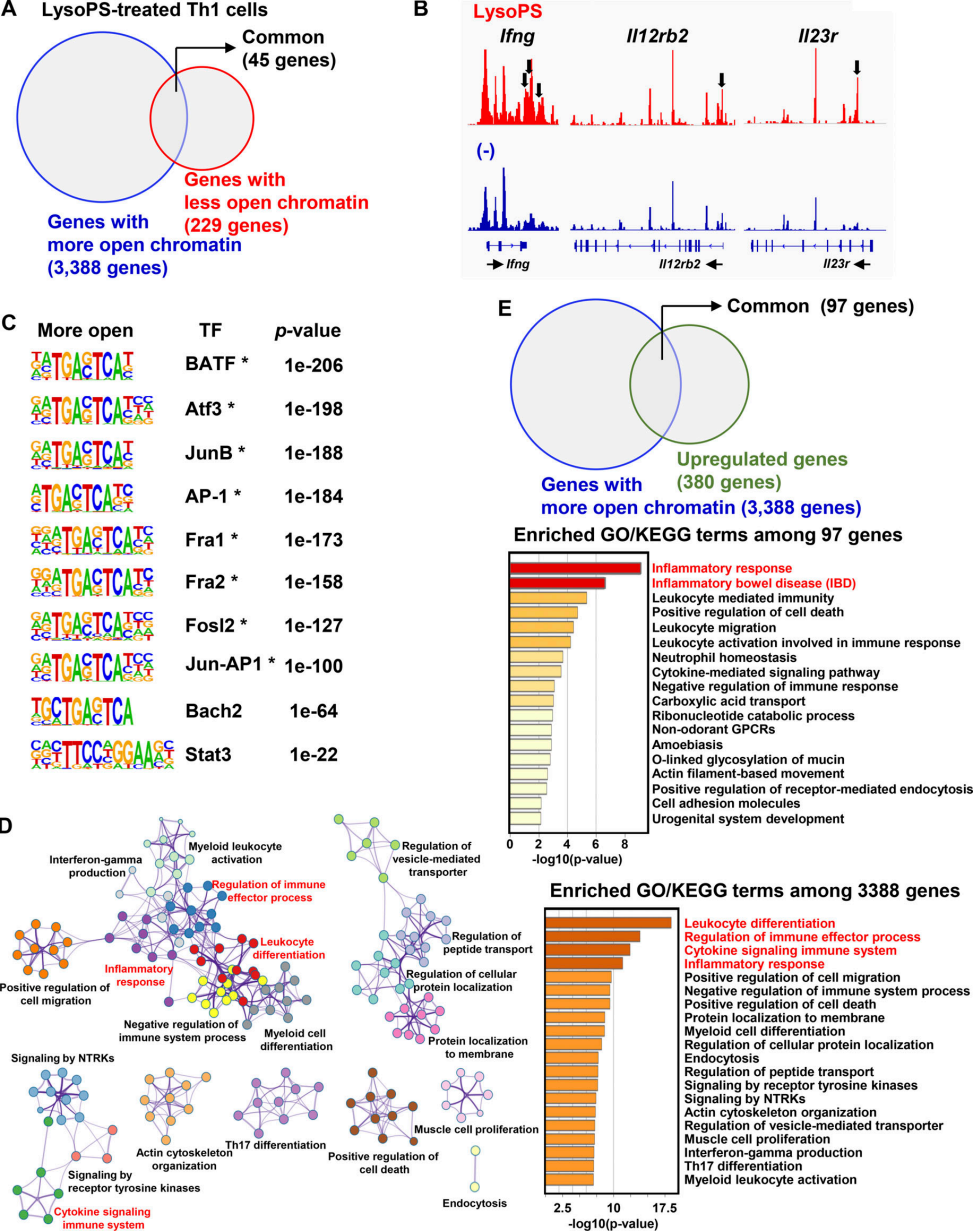
**LysoPS regulated transcriptome programs of IFN-γ–producing CD4**^**+**^ **T cells. (A)** The numbers of genes with more (blue) or less (red) open chromatin regions. **(B)** The Integrative Genomics Viewers snapshot shows the ATAC-seq signal at three representative loci (*Ifng*, *Il12rb2*, and *Il23r*). Arrows indicate more accessible regions in LysoPS-stimulated cells. **(C)** Top 10 DNA-binding motifs enriched in the more open chromatin regions of LysoPS-treated cells. The asterisk indicates an AP-1 motif. **(D)** Left: Enrichment network visualizations of GO and KEGG. Right: Bar graphs of nonredundant GO/KEGG enriched terms. **(E)** Upper: Venn diagram showing the gene overlap among genes upregulated by LysoPS in the RNA-seq analysis and ATAC-seq peak. Lower: Heatmap of enriched GO/KEGG terms among the 97 common genes.

The TCA cycle metabolites regulate not only proliferation or survival in effector T cells but also transcription, via chromatin modification (Martínez-Reyes and Chandel, 2020; Sivanand et al., 2018). Therefore, we examined whether LysoPS affects chromatin structure by performing an assay for transposase-accessible chromatin with sequencing (ATAC-seq) analysis (Fig. 7). In Th1-skewed cells, the number of genes (3,388 genes) that acquired more open chromatin regions (±20 kb from TSS) in response to LysoPS was markedly higher than that of genes with reduced chromatin accessibility (229 genes; Fig. 7 A and Tables S5 and S6). Notably, the genes with increased accessibility included key genes relevant to T-cell effector functions, such as *Ifng*, *Il12rb2*, and *Il23r* (Fig. 7 B). A HOMER analysis identified activator protein 1 (AP-1) motifs as transcription factor (TF)–binding sites overrepresented within more accessible chromatin regions in LysoPS-treated Th1 cells (Fig. 7 C). This finding was in accordance with a previous study showing AP-1–binding motif enrichment within more open chromatin regions in activated human CD4^+^ T cells (Yukawa et al., 2020). A Metascape enrichment analysis (http://metascape.org; Zhou et al., 2019) of genes with more accessible regions revealed several enriched terms and their networks (Fig. 7 D). Enriched processes with high significance were related to leukocyte differentiation, immune effector process regulation, cytokine signaling immune system, and inflammatory responses in Th1-skewed cells. We further analyzed whether increased chromatin accessibility correlated with transcriptome profile alterations in LysoPS-treated Th1 cells through a cross-comparison between more open chromatin genes and upregulated genes identified by ATAC-seq and RNA-seq analyses, respectively (Fig. 7 E). Among the 380 genes that were transcriptionally upregulated by LysoPS (Table S7), 97 possessed more accessible chromatin regions in in vitro–generated Th1 cells (Table S8). Moreover, this subset was enriched for genes predicted to associate with inflammatory responses or inflammatory bowel disease (Fig. 7 E). These findings suggest that the induction of epigenetic changes is one mechanism by which LysoPS promotes Th1-cell effector functions.

### P2Y10 receptor mediated LysoPS responses of IFN-γ–producing CD4^+^ T cells

**Figure S4.**
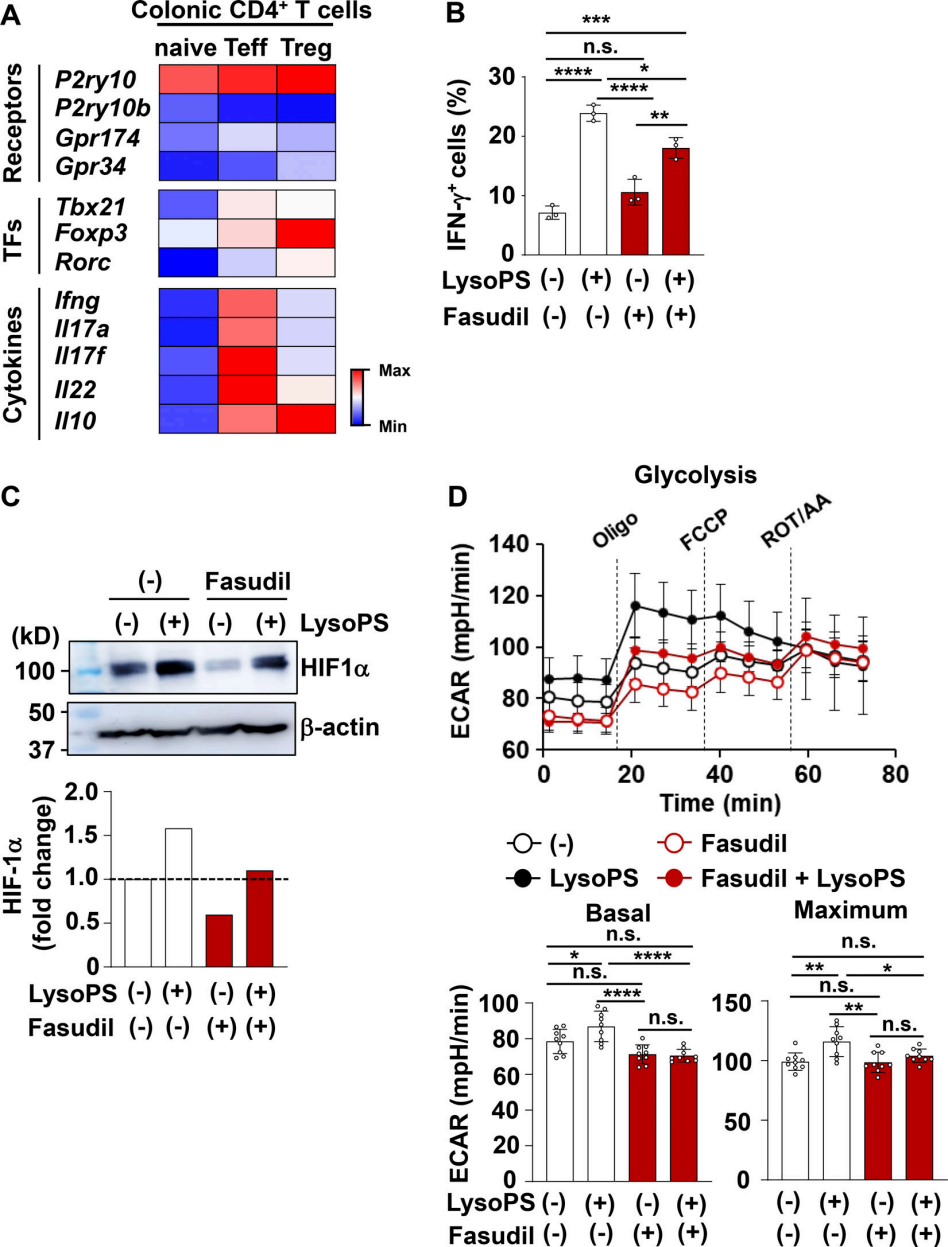
**LysoPS activated the Rho-ROCK signaling pathway in in vitro–polarized Th1 cells. (A)** Heatmaps of differentially expressed genes of LysoPS receptors, TFs, and cytokines in CD3^+^ CD4^+^ CD25^−^ CD62L^−^ CD44^+^ effector T cells, CD3^+^ CD4^+^ CD25^−^ CD44^−^ CD62L^+^ naive T cells, or CD3^+^ CD4^+^ CD25^+^ regulatory T cells from colonic lamina propria of C57BL/6J mice. **(B)** Percentage of IFN-γ–producing cells among CD4^+^ T cells cultured under Th1-polarized conditions with or without 10 µM 18:1 LysoPS in the presence or absence of 0.33 µM Fasudil (mean values ± SD from three independent experiments). *, P <0.05; **, P < 0.01; ****, P < 0.001. **(C)** Naive CD4^+^ T cells from mouse spleens were cultured under Th1-polarized conditions for 24 h, after which they were stimulated with 10 µM 18:1 LysoPS for 12 h and then used for immunoblotting with anti–HIF-1α and β-actin antibodies. Fold-change of HIF-1α protein was quantified using ImageJ. **(D)** Splenic naive CD4^+^ T cells were cultured under Th1-polarized conditions for 24 h, after which 10 µM 18:1 LysoPS was added into the culture with or without 0.33 µM Fasudil for 48 h. The basal and maximum ECAR were then analyzed in these cells. *, P < 0.05; **, P < 0.01; ****, P < 0.001. Graphs show mean values ± SD from at least seven wells. Data are representative of three independent experiments. All data were evaluated by one-way ANOVA. Source data are available for this figure: SourceData FS4.

**Figure S5.**
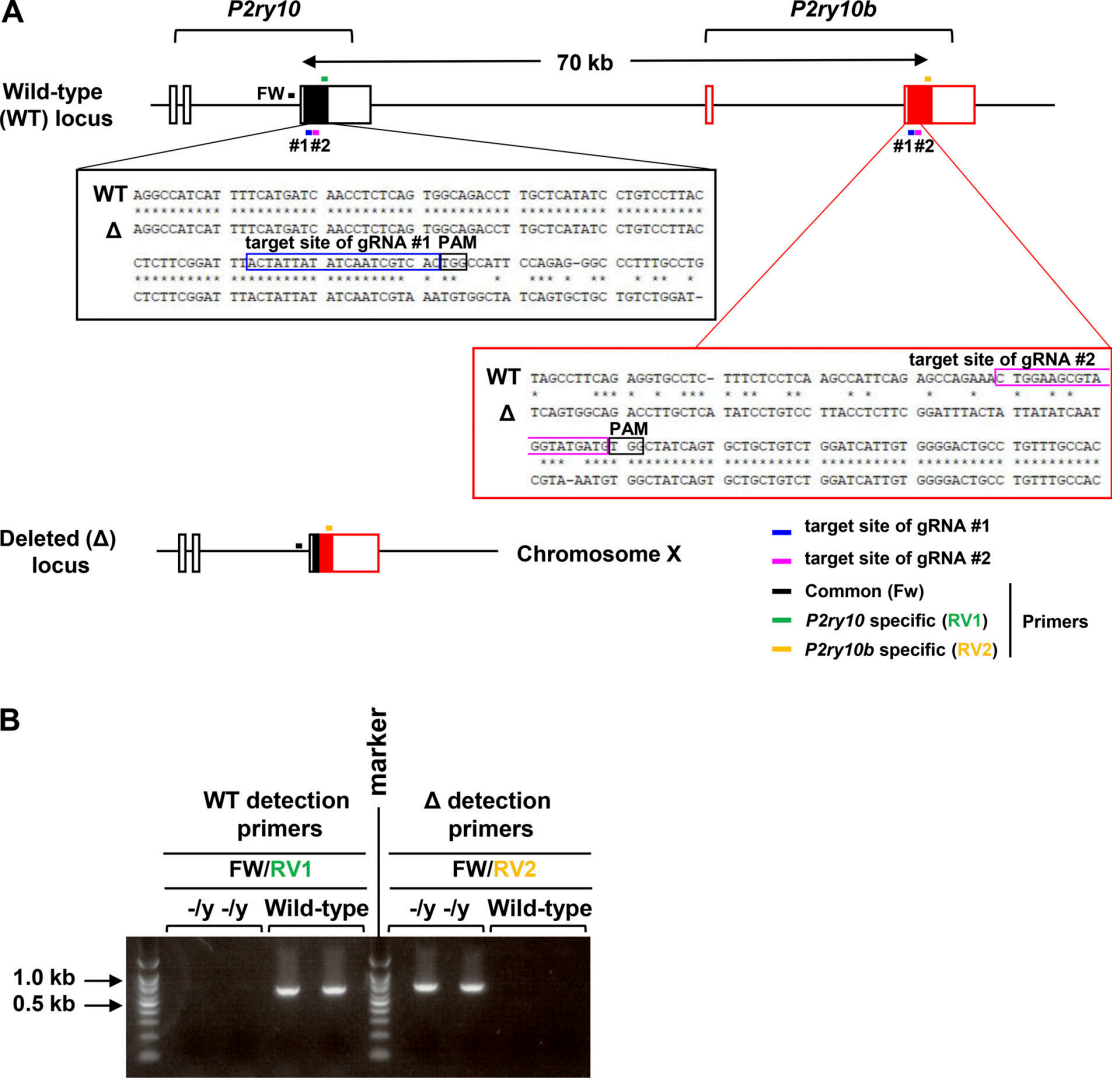
**Generation of *P2ry10*- and *P2ry10b*-deficient mice. (A)** Scheme of the Cas9/gRNA-targeting sites in the third exon of the *P2ry10* gene and the second exon of the *P2ry10b* gene (top). The sequences of the *P2ry10* and *P2ry10b* genes in the wild-type and mutated loci (middle). The structure of the *P2ry10* and *P2ry10b* genes in the deleted loci (bottom). Black boxes, coding exons; white boxes, noncoding exons. **(B)** PCR detection of male mice with wild-type or deleted loci. The primer sets used are indicated in A. Source data are available for this figure: SourceData FS5.

**Figure 8.**
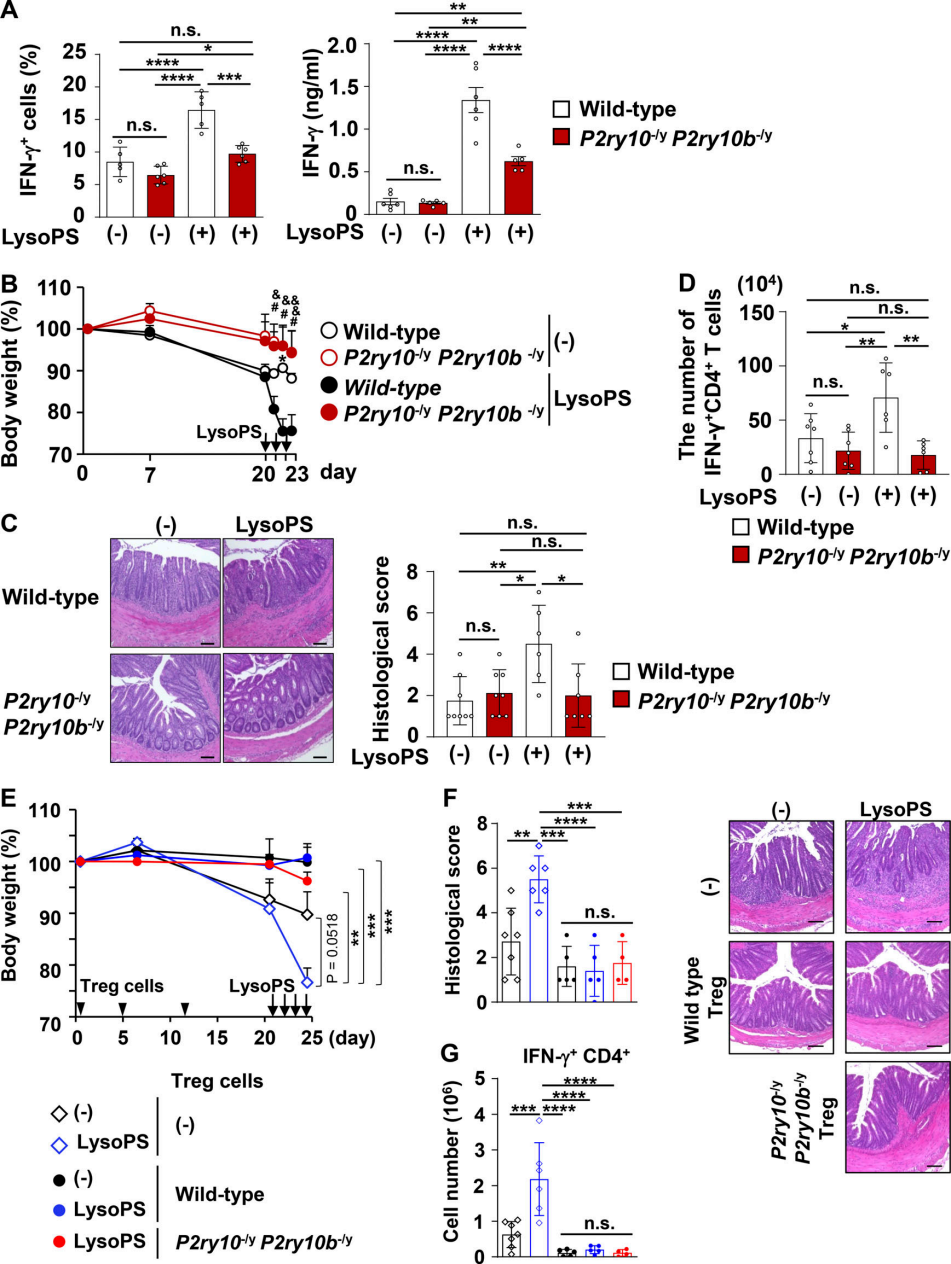
**P2Y10 mediated LysoPS-dependent activation of IFN-γ–producing CD4**^**+**^ **T cells. (A)** Left: Percentages of IFN-γ–producing CD4^+^ T cells (mean ± SD; wild-type, *n* = 5; mutant, *n* = 6). Right: Levels of IFN-γ secretion (mean ± SD; wild-type, *n* = 6; mutant, *n* = 5). Statistical analysis was performed via one-way ANOVA. *, P < 0.05; **, P < 0.01; ***, P < 0.005; ****, P < 0.001. Data were pooled from two independent experiments. **(B)** Body weight changes. *, P < 0.05; #, P < 0.05; &, P < 0.05 and &&, P < 0.01; *, P denotes a significant difference between untreated mice (*n* = 5) and LysoPS-treated mice that received wild-type cells (*n* = 4); #, P denotes a significant difference between LysoPS-treated mice that received wild-type cells and those that received *P2ry10*^−/y^*P2ry10b*^−/y^ cells (*n* = 5); and &, P denotes a significant difference between LysoPS-treated mice that received wild-type cells and untreated mice that received *P2ry10*^−/y^*P2ry10b*^−/y^ cells (*n* = 4). All data are from two independent experiments. **(C)** Left: Representative H&E-stained colon images. Right: Histological scores. Mean values ± SD from at least six mice/group. Data were pooled from two independent experiments. *, P < 0.05; **, P < 0.01. **(D)** The number of IFN-γ–producing CD4^+^ T cells in the colonic lamina propria. Data were pooled from two independent experiments and show mean values ± SD from at least six mice/group. *, P < 0.05; **, P < 0.01. All data were evaluated by one-way ANOVA. **(E)** Body weight changes of *Rag2*^−/−^ mice that received intraperitoneal injection of LysoPS (non-cotransfer of Treg cells, *n* = 6; cotransfer of wild-type Treg cells, *n* = 5; and cotransfer of *P2ry10*^−/y^*P2ry10b*^−/y^ Treg cells, *n* = 4) or vehicle (non-cotransfer of Treg cells, *n* = 7; and cotransfer of wild-type Treg cells, *n* = 5). Data were pooled from two independent experiments and were evaluated by one-way ANOVA. **, P < 0.01; ***, P < 0.005. **(F)** Left: Representative H&E-stained colon images. Right: Histological scores (mean values ± SD from at least four mice/group). Data were pooled from two independent experiments. **, P < 0.01; ***, P < 0.005; ****, P < 0.001. **(G)** The number of IFN-γ–producing CD4^+^ T cells in the colonic lamina propria (mean values ± SD from at least four mice/group). Data were pooled from two independent experiments. ***, P < 0.005; ****, P < 0.001. Data were evaluated by one-way ANOVA. Scale bars: 100 μm.

We next attempted to identify the signaling pathway responsible for the LysoPS-dependent facilitation of Th1 responses. Previous studies have shown that LysoPS binds to G protein–coupled receptors, such as GPR34, GPR174, P2Y10, and P2Y10b (Kihara et al., 2014). Among these receptors, colonic CD4^+^ T cells, including naive T cells, effector T cells, and Treg cells, highly express *P2ry10* (Fig. S4 A). P2Y10 has been demonstrated to activate Gα_12/13_ signaling, which elicits the Rho/Rho-associated kinase (ROCK) pathway (Kihara et al., 2014). To investigate whether P2Y10 mediates the LysoPS-induced activation of Th1 responses, naive CD4^+^ T cells were cultured under Th1-polarizing conditions with or without the ROCK inhibitor Fasudil. The LysoPS-dependent induction of IFN-γ–producing cells in Th1 culture was reduced by Fasudil (Fig. S4 B). Fasudil can reduce the protein level of HIF-1α by inhibiting the Rho-ROCK signaling pathway (Τakata et al., 2008). Here, HIF-1α protein was increased in in vitro–polarized Th1 cells in response to LysoPS (Fig. S4 C), and this effect was impaired in Fasudil-treated cells. We further analyzed the effect of LysoPS-dependent ROCK signaling pathway activation on Th1 cell metabolic changes. Fasudil suppressed the LysoPS-induced elevation of basal and maximum ECAR in Th1-skewed cells (Fig. S4 D), which suggests that LysoPS accelerates Th1 responses by activating the Rho-ROCK signaling pathway via the P2Y10 receptor. However, the function of P2Y10 receptor remains poorly understood. Because *P2ry10b*, located nearby on chromosome X, shows a high degree of homology with *P2ry10*, we analyzed mice lacking both *P2ry10* and *P2ry10b* (*P2ry10*^−/y^*P2ry10b*^−/y^ mice; Fig. S5, A and B). To evaluate whether the lack of *P2ry10* and *P2ry10b* influences LysoPS-dependent CD4^+^ T cell responses, naive CD4^+^ T cells isolated from the spleens of wild-type and *P2ry10*^−/y^*P2ry10b*^−/y^ mice were cultured under Th1-polarizing conditions with or without LysoPS (Fig. 8 A). In the absence of LysoPS, deficiency of *P2ry10* and *P2ry10b* did not affect the percentage of IFN-γ–producing cells or IFN-γ secretion level in cultured CD4^+^ T cells. However, the LysoPS-induced elevations in IFN-γ–producing cell percentage and IFN-γ secretion level that were observed in wild-type CD4^+^ T cells were dramatically lower in *P2ry10*- and *P2ry10b*-deficient cells. Because lower LysoPS responsiveness was observed in *P2ry10*^−/y^*P2ry10b*^−/y^ T cells, we assessed the impact of *P2ry10* deficiency in CD4^+^ T cells on LysoPS-induced intestinal pathology. Naive CD4^+^ T cells from the spleens of wild-type or *P2ry10*^−/y^*P2ry10b*^−/y^ mice were transferred into *Rag2*^−/−^ mice that were intraperitoneally injected with LysoPS at the indicated time points. LysoPS-untreated *Rag2*^−/−^ mice that received *P2ry10*^−/y^*P2ry10b*^−/y^ naive T cells showed a slightly reduced body weight loss compared with mice that received wild-type naive T cells (Fig. 8 B). LysoPS drastically enhanced body weight loss and worsened large intestinal pathology in *Rag2*^−/−^ mice that received wild-type naive T cells but not in mice that received *P2ry10*^−/y^*P2ry10b*^−/y^ naive T cells (Fig. 8, B and C). In this context, LysoPS increased the number of IFN-γ^+^ CD4^+^ T cells in the large intestinal lamina propria of *Rag2*^−/−^ mice that received wild-type cells (Fig. 8 D), whereas it did not affect the number of these cells in mice that received *P2ry10*^−/y^*P2ry10b*^−/y^ CD4^+^ T cells.

Because colonic Treg cells expressed *P2ry10* (Fig. S4 A) and LysoPS affected glycolytic metabolism in in vitro–skewed Treg cells (Fig. 6 F), we analyzed the impact of LysoPS on the suppressive activity of Treg cells in this colitis model (Fig. 8, E–G). *Rag2*^−/−^ mice were transferred wild-type naive T cells with or without CD4^+^CD25^+^ Treg cells from the spleens of wild-type or *P2ry10*^−/y^*P2ry10b*^−/y^ mice. Cotransfer of wild-type Treg cells dramatically reduced LysoPS-mediated weight loss and intestinal pathology in *Rag2*^−/−^ mice transferred naive T cells (Fig. 8, E and F). In addition, the number of IFN-γ^+^ CD4^+^ T cells in the colon of LysoPS-treated *Rag2*^−/−^ mice was markedly decreased by wild-type Treg cell coadministration (Fig. 8 G). Coadministration of *P2ry10*^−/y^*P2ry10b*^−/y^ Treg cells also prevented LysoPS-mediated aggravation of colitis, as evidenced by decreases in body weight loss and histopathological score, in *Rag2*^−/−^ mice that received naive CD4^+^ T cells. In addition, *P2ry10*^−/y^*P2ry10b*^−/y^ Treg cells decreased the number of IFN-γ^+^ CD4^+^ T cells in the colon of LysoPS-treated *Rag2*^−/−^ mice to similar to that observed in mice cotransferred wild-type Treg cells with naive CD4^+^ T cells. Thus, although LysoPS influenced metabolic states of Treg cells, it did not affect Treg functions in vitro and in vivo (Figs. S3 D and 8 G). Taken together, these findings indicate that P2Y10 receptor activation by LysoPS elicits immunopathological Th1 responses with a Treg cell–independent mechanism, which leads to the aggravation of intestinal inflammation.

## Discussion

In this study, we revealed the mechanism by which dysbiosis induces aberrant Th1 responses and thereby promotes intestinal inflammation in CD. We showed that LysoPS confers an immunopathological profile to IFN-γ–producing CD4^+^ T cells through P2Y10 receptor–mediated metabolic reprogramming, which leads to colitis aggravation.

Patients with CD are shown to harbor dysbiotic microbial communities, associated with alterations of metabolite concentrations in sera and feces (Lavelle and Sokol, 2020). Similar to a previous study showing an altered fecal metabolomic profile using LC-MS (Franzosa et al., 2019), the present study uses a specific UPLC-ESI-MS/MS method and shows that phosphatidylcholine (PC) concentration is elevated in the stool samples of patients with CD. Concentration of PC, a major phospholipid in mucus, is shown to be lower in the intestinal lumen of patients with ulcerative colitis (UC) than those with CD and healthy individuals (Braun et al., 2009; Ehehalt et al., 2004). In addition, PC treatment achieved the higher remission rate in patients with UC compared with placebo treatment (Torres et al., 2013), suggesting that appropriate amounts of PC in the intestinal lumen are required for barrier integrity through establishment of hydrophobic surface of epithelial cell layers. However, the influence of excess PC generation in patients with CD remains unknown. Thus, it would be an important issue to clarify the impact of PC on regulation of intestine inflammation in CD.

CD is a multifactorial disease, caused by dysbiosis and abnormal environmental factors as well as genetic variations, and it remains poorly understood how dysbiosis leads to intestinal inflammation. We showed that dysbiotic microbiota facilitates LysoPS production in the intestine of some patients with CD. Moreover, LysoPS promoted pathological Th1 responses and thereby aggravates colitis in mouse models. Previous studies suggest the involvement of *E. coli* in CD pathogenesis. *E. coli* expansion in the intestine of patients with CD has been shown (Chassaing et al., 2011; Darfeuille-Michaud et al., 1998; Nagayama et al., 2020), and a *E. coli* strain that was increased in patients with CD induced accumulation of Th1 cells in mouse intestine (Nagayama et al., 2020). Colonization of adherent-invasive *E. coli* was reported to aggravate intestinal inflammation (Carvalho et al., 2009; Palmela et al., 2018). Elevation of LysoPS concentration has been shown in the intestine of gnotobiotic mice colonized with *E. coli* compared with germ-free mice (Chakrabarti et al., 2017). Thus, the enhanced production of LysoPS in the intestine due to the colonization of increased numbers of *E. coli* might be one of mechanisms by which dysbiosis enhances pathological Th1 responses in the intestine, thereby promoting the progression of CD.

Lipid species elevated in the plasma of CD patients (Iwatani et al., 2020) would be generated by host enzymes of nonintestinal tissues in the inflammatory states. Among them, concentrations of 18:0 LysoPS and 18:1 LysoPS were elevated in both the plasma and feces of some patients with CD, suggesting that LysoPS produced by dysbiotic microbiota in the intestine enters into the circulation. Serum concentration of 18:1 LysoPS was ∼1.7-fold higher in C57BL/6J mice injected with 18:1 LysoPS (2.5 mg/kg) intraperitoneally for 4 d than in control mice (vehicle, 0.92 ± 0.28; LysoPS, 1.56 ± 0.34 pmol/ml, P = 0.11), which was comparable to the level of the increase in patients with CD (∼1.5-fold higher) relative to HCs (Fig. 1 E), indicating that the dose of 18:1 LysoPS used in the current study represents the upregulated level of 18:1 LysoPS in patients with CD.

Consistent with previous findings showing that glycolysis promotion in effector T cells is a cause of severe colitis (Macintyre et al., 2014), LysoPS exaggerates large intestinal inflammation by fueling glycolysis in IFN-γ–producing CD4^+^ T cells. In effector T cells, HIF-1α induces expression of glycolytic genes (Bantug et al., 2018). LysoPS stimulation increased the HIF-1α protein level in Th1-polarized cells. Therefore, LysoPS might activate glycolysis by accelerating HIF-1α–dependent transcription. In accordance with a previous study showing that complete inhibition of glycolysis by 2DG impaired the differentiation of human Foxp3^+^ Treg cells and their suppressive activity (De Rosa et al., 2015), 2DG inhibited differentiation of murine Foxp3^+^ Treg cells in vitro (2DG, 7.8 ± 0.54%; vehicle, 42.4 ± 0.54%, P = 0.0001: mouse naive CD4^+^ T cells were cultured in a Treg-polarizing condition in the presence or absence of 2DG). In contrast, LysoPS only partially inhibited glycolytic activity of Foxp3^+^ Treg cells, and therefore the induction and regulatory function of Treg cells might not be affected.

Under steady-state conditions, 18:0 LysoPS and 18:1 LysoPS are present at detectable levels in the spinal cord and colon (Barnes et al., 2015). A reduction of regulatory T cell activities by the LysoPS–GPR174 axis is implicated in the pathogenesis of experimental autoimmune encephalomyelitis (Barnes et al., 2015). In *Gpr174*-deficient mice, the number of CD103^+^ tissue-resident Foxp3^+^ Treg cells was increased in the spleen and lymph nodes, but not the thymus and colon, and promotion of the suppressive activity of Foxp3^+^ Treg cells was shown in the spleen. In this study, we showed that expression of *Gpr174* mRNA in colonic Treg cells was low and that LysoPS did not have any effect on the Treg cell-mediated suppression of colitis. Thus, LysoPS-GPR174 signaling might control differentiation and regulatory function of Foxp3^+^ Treg cells in the tissues other than the colon.

We observed lower *Gpr174* expression in colonic Treg cells, and *P2ry10* and *P2ry10b* deficiency in CD4^+^ T cells prevents LysoPS-dependent colitis exacerbation. Moreover, cotransfer of splenic Treg cells from wild-type or *P2ry10*^−/y^*P2ry10b*^−/y^ mice prevented LysoPS-mediated exaggeration of large intestinal inflammation in *Rag2*^−/−^ mice that received wild-type naive CD4^+^ T cells. Thus, we reason that LysoPS does not affect Treg cell function, at least in the colon.

LysoPS concentration is higher in ascites from patients with gastric cancer than in those with cirrhosis (Emoto et al., 2017). *P2RY10* expression is increased in the synovial tissue and peripheral blood from patients with rheumatoid arthritis and coronary artery disease, respectively (Wang et al., 2016). In addition, the LysoPS–P2Y10 axis has been shown to suppress TNF-α production in murine microglia and activate eosinophil degranulation (Hwang et al., 2018; Kita et al., 2019). In the present study, we proved that LysoPS facilitates Th1-mediated intestinal pathology via the P2Y10 receptor. Thus, LysoPS may possess a possible risk thatpromotes intestinal inflammation through immunomodulatory activities in the local tissues.

In conclusion, our study provides evidence for the contribution of LysoPS to intestinal inflammation progression via perturbations of host adaptive immunity. Regulation of lipid metabolites, including leukotrienes and prostaglandins, is an emerging therapeutic approach for inflammatory disorders (Serhan et al., 2014). Thus, the bioactive lipid LysoPS could be a putative diagnostic biomarker and a therapeutic target for CD.

## Materials and methods

### Human fecal samples

To perform whole-genome shotgun-seq shown in Fig. 2, A–C, fecal samples were collected from 40 HCs and 43 patients with CD (Table S9). We also obtained fecal samples from 12 HCs and 11 patients with CD shown in Fig. 1, and 3 patients with CD shown in Fig. 6, G and H, for conducting lipidomic analysis and T cell metabolic analysis (Table S10). CD was diagnosed in accordance with the established diagnostic criteria (Matsuoka et al., 2018). These studies were approved by the Institutional Research Ethics Board at Osaka University Hospital (15298-7). We obtained written informed consent from all patients and HC to use their samples and data.

### Mice

C57BL/6J mice were purchased from Clea Japan or Japan SLC. *Rag2*^−/−^ mice with a C57BL/6J background were purchased from Taconic Biosciences or The Jackson Laboratory. Tsl:ICR germ-free mice were purchased from SANKYO LABO SERVICE. 8–15-wk-old mice were used for the experiments. All mice were maintained under specific pathogen–free conditions or germ-free conditions until transplanted with human microbiota. All animal experiments were conducted in accordance with the guidelines of the Animal Care and Use Committee of Osaka University (28-025 and 28-065).

### Colonization of human fecal microbiota

The colonization of human fecal microbiota into germ-free mice was performed as described previously, with slight modification (Maeda et al., 2016). Fresh fecal samples were collected from four HCs with healthy microbiota or two patients with CD harboring microbiota with an abundance of *ECSF_3660* and mixed. The fecal samples were homogenized in an aerobic buffer (2% Lab-Lemco powder, 0.1% L-cysteine, 0.045% KH_2_PO_4_, 0.09% NaCl, 0.045% [NH_4_]_2_SO_4_, 0.045% CaCl_2_, 0.045% MgSO_4_, and 40% glycerol in 1,000 ml) at a 16-fold dilution (wt/vol) and stored at −80°C until use. Male germ-free mice (8–9 wk old) were orally inoculated with 250 μl of the fecal suspensions from HCs or patients with CD, and they were maintained separately in gnotobiotic isolators for 24 d.

### Generation of *P2ry10*- and *P2ry10b*-deficient mice

To generate *P2ry10-* and *P2ry10b-*deficient mice via the Cas9/CRISPR system, a mixture of CRISPR RNAs (5′-ACT​ATT​ATA​TCA​ATC​GTC​AC-3′ for target sites of gRNA #1 and 5′-CTG​GAA​GCG​TAG​GTA​CGA​TG-3′ for target sites of gRNA #2; Sigma-Aldrich), trans-activating crRNA (Sigma-Aldrich), and Cas9 ribonucleoprotein (Sigma-Aldrich) was injected into the pronuclei of one-cell-stage embryos from C57BL/6J mice with an electroporator (Nepagene). These eggs were cultivated in KSOM (Ark Resource) for 20 h and then transferred into the oviducts of pseudo-pregnant females anesthetized via intraperitoneal injection with a mixture of three types of anesthetic agents (0.003% medetomidine, 0.04% midazolam, and 0.05% butorphanol tartrate). Germline transmission was confirmed by mating the obtained chimeric mice with C57BL/6J mice. To search for gRNA and off-target sequences, we used CRISPRdirect software (https://crispr.dbcls.jp/) and Benchling (https://www.benchling.com/crispr/). The following primer sets, denoted as shown in Fig. S5, were used for genotyping: 5′-TAT​TTA​ACC​ATT​GTG​CCT​TTA​AAC​TTT​GTG-3′ (common Fw) and 5′-GGT​GAC​CAG​AAC​CAC​TGC​ATC​CAT​CTG​TTT​G-3′ (*P2ry10*-specific [RV1]) for wild-type locus detection and 5′-TAT​TTA​ACC​ATT​GTG​CCT​TTA​AAC​TTT​GTG-3′ (common Fw) and 5′-GGG​AAG​TTG​AGA​TGG​TAA​GG-3′ (*P2ry10b*-specific [RV2]) for deleted locus detection. The amplification conditions were 94°C (5 min), followed by 35 cycles of 94°C (30 s), 59°C (60 s), and 72°C (60 s). Mice of all genotypes were born at the expected frequencies.

### Reagents

Etomir, UK5099, BPTES, the Ca^2+^ ionophore A23187, PMA, TNBS, 2DG, and the Gene Elute Mammalian Total RNA Miniprep kit were purchased from Sigma-Aldrich. DSS (molecular weight 36–50 kD) was purchased from MP Biomedicals. 18:0 LysoPSa, 18:1 LysoPSa, and 18:1 LysoPCa were purchased from Avanti Polar Lipids. [^3^H]thymidine was purchased from PerkinElmer Japan. Fasudil was purchased from Tokyo Chemical Industry. Dormicum (Midazolam) was purchased from Astellas. Butorphanol tartrate was purchased from Meiji Seika Pharma. Domitor (Medetomidine) was purchased from Zenoaq. The Seahorse XF Cell Mito Stress Test Kit, Seahorse XF RPMI Medium, Seahorse XF Glucose, Seahorse XF Pyruvate, and Seahorse XF L-Glutamine were purchased from Agilent Technologies. Corning Cell-Tak Cell and Tissue Adhesive was purchased from Corning. Percoll was purchased from GE Healthcare. Ficoll-Paque PREMIUM was purchased from Cytiva. RNAlater was purchased from Ambion. Glass beads (diameter 0.1 mm) were purchased from BioSpec Products. The ROS Assay Kit (Deep Red) was purchased from Abcam. The ATAC-seq Kit was purchased from Active Motif. Precision Plus Protein All Blue Standards were purchased from Bio-Rad Laboratories. AccuRuler 100-bp DNA RTU Ladder was purchased from MaestroGen. Mouse IFN-γ CBA Flex Set, anti-mouse CD16/CD32 (clone 2.4.G2), anti-mouse CD3e-Pe/Cy7 (145-2C11), anti-mouse IL-17A-Alexa Fluor 647 (TC11-18H10), anti-mouse CD3e (145-2C11), anti-mouse CD28 (37.51), and anti-mouse CD25 (7D4) antibodies were purchased from BD Biosciences. CD4 (L3T4) MicroBeads, CD11c MicroBeads UltraPure, and Naive CD4^+^ T cell Isolation Kit were purchased from Miltenyi Biotec. Anti–HIF-1α antibody was purchased from Novus Biologicals. Anti-mouse CD4-PerCP/Cy5.5 (GK1.5), anti-mouse IL-10-PE (JES5-16E3), anti-mouse IFN-γ -FITC (XMG1.2), anti-mouse CD4-Pacific blue (GK1.5), anti-mouse CD62L-PerCP/Cyanine5.5(MEL-14), anti-mouse IL-22-APC (Poly5164) antibodies, anti-human CD4-APC (SK3), anti-human CD45RA-Brilliant Violet 421 (HI100), anti-human CD4-Pacific Blue (RPA-T4) or APC (SK3), anti-human IFN-γ-APC (4S.B3), anti-human CD45RO-FITC (UCHL1), anti-human CD25-APC/Cyanine7 (BC96) antibodies, and 7-aminoactinomycin D were purchased from BioLegend. Anti-mouse IL-4 (11B11), anti-mouse IFN-γ (XMG1.2), and anti-human IL-4 (MP4-25D2) antibodies were purchased from eBiosciences. Recombinant murine IL-12 (p70), recombinant human IL-12 (p70), and recombinant human TGF-β1 were purchased from Peprotech. Recombinant murine IL-6, recombinant murine IL-1β, recombinant murine IL-23, and anti-mouse IL-2 (JES6-1A12) antibodies were purchased from R&D Systems. Anti-Hu/Mo CD44 (IM7) antibody was purchased from Invitrogen. Anti-human CD3/CD28 beads were purchased from Gibco. LysoPS and LysoPC were dissolved in 70% ethanol at a concentration of 10 μM and stocked at −20°C. 70% ethanol was used as a vehicle control.

### Fecal lipidomic analysis

The concentrations of lipid molecules in fecal samples were measured as described previously (Muranaka et al., 2017) with slight modifications. In brief, frozen fecal samples were crushed, and several pieces were homogenized in methanol containing 1% acetic acid (100 μl per 10 mg of sample) and then sonicated on ice for 60 s. A 100-μl aliquot of each resulting homogenate was mixed with 2 ml of a mixture of chloroform, methanol, and ethanol (1:2:2 ratio) containing the internal standards (Table S11) and 100 μl of a mixture of chloroform, methanol, ethanol, and acetic acid (2:1:1:1 ratio) and centrifuged for 10 min at 4°C. The organic layer was collected and concentrated via evaporation with an EZ-2 Plus Genevac centrifugal evaporator (SP Scientific), and the dried contents were dissolved in 280 μl of a mixture of chloroform, methanol, and ethanol (1:2:2 ratio) as a reconstitution solution, of which 4 μl was used in the UPLC-ESI-MS/MS in the laboratory of Ono Pharmaceutical Company to analyze fecal lipid molecular species. We measured lipid concentrations twice per fecal sample from each person, and the average of these concentrations was used for subsequent analyses. Data were analyzed using MetaboAnalyst 5.0 (https://www.metaboanalyst.ca/MetaboAnalyst/ModuleView.xhtml) with the default settings. Data regarding the concentrations of 18:0 LysoPS and 18:1 LysoPS in the plasma from HCs and patients with CD were modified from the previous publication (Iwatani et al., 2020).

### Colitis induction

The TNBS-induced colitis model was generated as described previously (Iijima et al., 2004) with slight modifications. In brief, male C57BL/6J (8–10-wk-old) mice were sensitized with 150 μl of 3.75% TNBS in 50% ethanol by skin painting, and 7 d later, these mice were given an intrarectal injection of 150 μl of 2% TNBS in 50% ethanol and intraperitoneally administered LysoPS (2.5 mg/kg) or vehicle for 4 d consecutively. The intestines of these mice were collected 24 h after the last LysoPS injection. In some of the experiments, mice received an intrarectal injection of LysoPS (10 mg/kg) or vehicle with TNBS, and the intestines of these mice were collected 96 h after the LysoPS injection.

To generate the DSS-induced colitis model, male C57BL/6J mice (8–10 wk old) were orally administered 2.0% DSS dissolved in their drinking water and concomitantly injected intraperitoneally with LysoPS (2.5 mg/kg) once per day for 9 d. The intestinal tissues of these mice were isolated 10 d after initiation of DSS administration.

Naive CD4^+^ T cells (5 × 10^5^) magnetically isolated from the spleens of C57BL/6J mice were transferred into *Rag2*^−/−^ mice (8–15 wk old; Taconic Biosciences) by an intraperitoneal injection; 17 d later, LysoPS (2.5 mg/kg) was intraperitoneally administered once per day to these mice for 4 d (Fig. 4). The colons of these mice were collected 24 h after the last LysoPS injection. Naive CD4^+^ T cells (4 × 10^5^) isolated from the spleens of wild-type or *P2ry10*^−/y^*P2ry10b*^−/y^ mice with a FACSAria flow cytometer were transferred into *Rag2*^−/−^ mice (8–15 wk old; The Jackson Laboratory) by an intraperitoneal injection; 23 d later, LysoPS (2.5 mg/kg) was intraperitoneally administered once daily to these mice for 3 d (Fig. 8, A–D). In Fig. 8, E–G, naive CD4^+^ T cells (5 × 10^5^) magnetically isolated from the spleens of C57BL/6J mice were transferred into *Rag2*^−/−^ mice (8–15 wk old; The Jackson Laboratory) intraperitoneally, and some groups also received CD4^+^ CD25^+^ regulatory T cells (2 × 10^5^) from spleens of wild-type or *P2ry10*^−/y^*P2ry10b*^−/y^ mice 1, 5, and 12 d after naive T cell transfer; 21 d later, LysoPS (2.5 mg/kg) was intraperitoneally administered to these mice for 4 d. The colons of these mice were harvested 24 h after the last LysoPS injection. The severity of colitis was evaluated by analyzing body weight change, colon length, and large intestinal histopathology.

### Histopathological analysis

The colons of mice were fixed in formaldehyde or 4% paraformaldehyde and embedded in paraffin. Paraffin-embedded sections mounted on glass slides (4-μm thickness) were used for H&E staining, and images of H&E staining were acquired using Biozero (BZ9000; Keyence) or BX53 (Olympus). Each colon sections were evaluated using the inflammation scores for TNBS-induced colitis (Iijima et al., 2004), DSS-induced colitis (Dohi et al., 2005), or adoptive-transfer colitis (Liu et al., 2000), as described previously.

### Cell isolation

Murine CD4^+^ CD25^−^ CD44^−^ CD62L^+^ naive CD4^+^ T cells were isolated from the spleen with a FACSAria flow cytometer (BD Biosciences) or magnetically isolated with a naive CD4^+^ T cell Isolation Kit, and CD4^+^ CD25^+^ regulatory T cells were isolated from the spleen with a FACSAria flow cytometer. To collect human peripheral blood mononuclear cells (PBMCs), peripheral blood was suspended with an equal volume of PBS containing 2% FBS and overlaid on Ficoll in a 15-ml tube, and the separation of PBMCs was performed through centrifugation 500 *g* for 20 min at room temperature. PBMCs at the interface between Ficoll and PBS containing 2% FBS were collected and washed with PBS, after which CD4^+^ CD45RA^+^ naive cells or CD4^+^ CD25^−^ CD45RO^+^ memory effector T cells were isolated from the PBMCs with a FACSAria flow cytometer.

For the isolation of adaptive lymphocytes from the large intestinal lamina propria, a previously described protocol was used with slight modifications (Atarashi et al., 2008). In brief, the large intestines were longitudinally opened and washed with PBS to remove the feces and then placed in HBSS with 5 mM EDTA. After being incubated at 37°C for 20 min in a shaking water bath, the intestines were washed in PBS and cut into small pieces, which were then incubated in RPMI 1640 containing 4% FBS, 1 mg/ml collagenase D (Roche), 0.5 mg/ml dispase (Thermo Fisher Scientific), and 40 μg/ml DNase I (Sigma Aldrich) for 35 min at 37°C in a shaking water bath. The digested tissues were resuspended in 5 ml of 40% Percoll and overlaid on 2.5 ml of 80% Percoll in a 15-ml tube. Percoll gradient separation was performed through centrifugation at 780 *g* for 20 min at room temperature. The lamina propria lymphocytes at the interface of the Percoll gradient were collected and washed with PBS containing 2% FBS, after which CD3^+^ CD4^+^ CD25^−^ CD62L^−^ CD44^+^ effector T cells, CD3^+^ CD4^+^ CD25^−^ CD44^−^ CD62L^+^ naive T cells, or CD3^+^ CD4^+^ CD25^+^ Treg cells were isolated with a FACSAria flow cytometer in some experiments.

### Induction of Th1-, Th17-, Treg-, or Th0-polarized cells

To induce IFN-γ–producing CD4^+^ T cells, murine splenic naive CD4^+^ T cells were cultured in 10 μg/ml anti-CD3 antibody–coated 24- or 48-well plates in the presence of 10 μg/ml anti–IL-4 antibody, 10 ng/ml IL-12, and 2 μg/ml anti-CD28 antibody; 24 h later, 18:1 or 18:0 LysoPS or 18:1 LysoPC was added to the culture at 10 μM unless otherwise stated. For RNA-seq, ATAC-seq, and metabolic assay, naive CD4^+^ T cells were cultured in 5 μg/ml anti-CD3 antibody–coated plates in the presence of the supplements described above. In some cases, an inhibitor such as UK5099 (10 μM), 2DG (350 μM), and Fasudil (0.33 μM) was added along with LysoPS. After 48 h, these cells were used for analyses. Human naive CD4^+^ T cells were cultured in the presence of anti-CD3/CD28 beads (1:2 ratio of beads:cells), 10 μg/ml anti–IL-4 antibody (eBioscience), and 10 ng/ml IL-12 (PeproTech) for 24 h; 18:1, 18:0 LysoPS, or 18:1 LysoPC was added to the culture with or without 2DG. 48 h later, these cells were analyzed for IFN-γ production or metabolic changes. For the induction of Th0 cells, murine splenic naive CD4^+^ T cells were cultured in 10 μg/ml anti-CD3 antibody–coated 24-well plates in the presence of 10 μg/ml anti–IL-4 antibody and 10 μg/ml anti–IFN-γ antibody. To induce Th17 cells, murine splenic naive T cells were cultured in 10 μg/ml anti-CD3 antibody–coated 24-well plates in the presence of 10 μg/ml anti–IL-4 antibody, 10 μg/ml anti–IFN-γ antibody, 1 μg/ml anti–IL-2 antibody, 2 μg/ml anti-CD28 antibody, 50 ng/ml IL-6, 2 ng/ml TGF-β, 10 ng/ml IL-1β, and 20 ng/ml IL-23; 24 h later, 10 μM 18:1 LysoPS was added to the culture for 48 h. To induce murine regulatory T cells, splenic naive T cells were cultured in 10 μg/ml anti-CD3 antibody–coated 24-well plates (for analysis of metabolic changes or Foxp3 expression) in the presence of 10 μg/ml anti–IL-4 antibody, 10 μg/ml anti–IFN-γ antibody, 2 μg/ml anti-CD28 antibody, and 5 ng/ml TGF-β; 24 h later, 10 μM 18:1 LysoPS was added to the culture for 48 h, and these cells were analyzed metabolic changes, suppressive activity, and Foxp3 expression.

### Cytokine analysis

Splenic CD4^+^ cells isolated with the MACS technology (Miltenyi Biotec) were cultured in the presence of 5 μg/ml anti-CD3 antibody and 5 μg/ml anti-CD28 antibody with LysoPS at a concentration of 0, 0.1, 1, or 10 μM for 24 h, and the IFN-γ levels in the supernatants were measured with an IFN-γ Mouse Uncoated ELISA Kit (eBioscience) in accordance with the manufacturer’s instructions. The concentrations of IFN-γ in the culture supernatants of wild-type or *P2ry10*^*−/y*^*P2ry10b*^*−*/y^ CD4^+^ T cells were determined by using Cytometric Bead Array kit (BD Biosciences). For intracellular cytokine staining, in vitro–polarized Th1 cells or colonic lamina propria adaptive lymphocytes were stimulated with 50 ng/ml PMA and 5 μM ionomycin in complete RPMI 1640 at 37°C for 4 h in the presence of GolgiStop (BD Biosciences). Surface staining was performed with anti-CD4 antibody at 4°C for 20 min, and intracellular cytokine staining was performed with anti–IL-10, anti–IL-17A, or anti–IFN-γ antibodies for 20 min by using a Cytofix/Cytoperm Kit Plus (BD Biosciences) in accordance with the manufacturer’s instructions. Flow cytometric analysis was performed with a FACSCanto II flow cytometer (BD Biosciences) or FACSAria (BD Biosciences) with FlowJo software (TreeStar). The instrumental compensation was set in each experiment using single-color or two-, three-, four-, or five-color stained samples.

### Quantitative RT-PCR

Total RNA was extracted using an RNeasy Mini Kit (Qiagen), and complementary DNA was synthesized using the ReverTra Ace qPCR RT Master Mix (Toyobo) in accordance with the manufacturer’s instructions. Real-time RT-PCR was performed using primers for *β-actin*, *Ifng*, *Il17a*, *Il23a*, *Il12b*, *Il22*, and *Il10* obtained from Applied Biosystems and THUNDERBIRD Probe qPCR mix (Toyobo) on an ABI Prism 7900HT sequence detection system (Applied Biosystems). All values were normalized to the expression of *β-actin*, and the fold difference in expression relative to that of *β-actin* is shown. The amplification conditions were 50°C (2 min), 95°C (10 min), and 45 cycles of 95°C (15 s) and 60°C (60 s). In some of the experiments, total RNA was isolated using the GenElute Mammalian Total RNA Miniprep Kit (Sigma-Aldrich), and the resulting RNA was reverse transcribed with ReverTra Ace qPCR RT Master Mix with gDNA Remover (Toyobo). Real-time RT**-**PCR was conducted on a Step One Plus Real-Time PCR System (Applied Biosystems) using Power SYBER Green PCR Master Mix (Applied Biosystems). The value of *Ifng* was normalized to the expression of *Gapdh*, which encodes GAPDH, and the fold difference in expression relative to that of *Gapdh* is shown. The amplification conditions were 50°C (2 min), 95°C (10 min), and 40 cycles of 95°C (15 s) and 60°C (60 s). The following primer sets were used: *Gapdh*, 5′-CCT​CGT​CCC​GTA​GAC​AAA​ATG-3′ and 5′-TCT​CCA​CTT​TGC​CAC​TGC​AA-3′; *Ifng*, 5′-TCA​AGT​GGC​ATA​GAT​GTG​GAA​GAA-3′ and 5′-TGG​CTC​TGC​AGG​ATT​TTC​ATG-3′.

### RNA-seq and ATAC-seq

Naive CD4^+^ T cells from the spleens of C57/BL6J mice were cultured under Th1-polarizing conditions for 24 h, and then 18:1 LysoPS or vehicle was added to the culture. After 48 h, a portion of these cells was stimulated with 50 ng/ml PMA and 5 μM ionomycin for 90 min. In addition, CD4^+^ naive, effector, and regulatory T cells were isolated from the colonic lamina propria of C57BL/6J mice using a FACSAria. Library preparation of in vitro–generated Th1 cells was performed using a TruSeq stranded mRNA sample prep kit (Illumina) in accordance with the manufacturer’s instructions. For library preparation of colonic CD4^+^ T cells, full-length cDNA was generated using a SMART-Seq HT Kit (Takara Bio). An Illumina library was prepared using a Nextera DNA Library Preparation Kit (Illumina) according to the SMARTer Kit instructions. Sequencing was performed on an Illumina NovaSeq 6000 platform for Th0 cells and on a HiSeq 2500 platform for Th1 cells, in 101- and 75-base single-end mode, respectively. The sequenced reads were mapped to the mouse reference genome sequences (mm10) using TopHat v2.0.13 in combination with Bowtie2 v2.2.3 and SAMtools v0.1.19. The fragments per kilobase of exon per million mapped fragments (FPKMs) were calculated using Cufflinks v2.2.1 (http://cole-trapnell-lab.github.io/cufflinks/). Heatmaps of the expression levels of receptors for LysoPS (Fig. S4 A) were generated from the FPKM values. BioJupies platform (https://amp.pharm.mssm.edu/biojupies/) with default settings provided a heatmap with the differential gene expression patterns among in vitro–polarized Th1 cells stimulated with or without LysoPS and PMA/ionomycin, as well as of the enriched pathways among in vitro–polarized Th1 cells genes upregulated in response to LysoPS regardless of PMA/ionomycin stimulation (Fig. 6 A). Among the calculated genes with a normalized FPKM value of >1.0 in LysoPS-treated and PMA/ionomycin-unstimulated cells, 380 genes were up-regulated >2.0-fold from their levels in LysoPS-untreated and PMA/ionomycin-unstimulated cells (see Table S7). For the ATAC reaction, 500,000 live cells among CD4^+^ T cells cultured under Th1-polarized conditions in the presence of 18:1 LysoPS or vehicle, as described above, were collected and centrifuged at 4°C at 800 *g* for 3 min and then washed once with RPMI. Libraries were prepared using the ATAC-seq Kit (Active Motif) in accordance with the manufacturer’s instructions. ATAC libraries were sequenced on an Illumina NovaSeq 6000 platform in 101-base single-end mode. After adapter trimming by Cutadapt v2.7, the trimmed reads were mapped to the mouse reference genome sequences (mm10) using Bowtie2 v2.3.5.1. Peak calling was performed with MACS2 v2.2.7.1 and visualized using Integrative Genomics Viewer (http://software.broadinstitute.org/software/igv/). For each gene, differential peaks were detected within ±20 kb around transcriptional start site. The enriched Gene Ontology (GO) and Kyoto Encyclopedia of Gene and Genomes (KEGG) pathways among genes with more open chromatin regions or among shared genes between genes possessing high-accessibility chromatin regions and transcriptionally upregulated in in vitro–polarized Th1 cells stimulated with LysoPS were identified using Metascape (https://metascape.org/gp/index.html#/main/step1).

### Metabolic assays

OCR and ECAR in in vitro–polarized Th1, Th17, Treg, or Th0 cells were analyzed with an XFe96 Extracellular Flux Analyzer (Seahorse Bioscience). Murine in vitro–generated Th1, Th17, Treg, or Th0 cells were collected 48 h after stimulation with 10 μM 18:1 LysoPS or 18:0 LysoPS, and 2 × 10^5^ of these cells were seeded onto Cell-Tak–coated XFe96 cell-culture microplates with Seahorse XF RPMI media supplemented 10 mM glucose, 1 mM pyruvate, and 2 mM L-glutamine (all from Agilent Technologies). The OCR and ECAR were measured under basal conditions and upon treatment with 1.5 μM oligomycin (Oligo), 2 μM fluorocarbonyl cyanide phenylhydrazone, and 0.5 μM rotenone/antimycinA. In some cases, an inhibitor, such as Etomoxir (4 μM), UK5099 (2 μM), or BPTES (3 μM), was added to each well just after the measurement of basal ECAR and OCR. 1.5 × 10^5^ of human blood CD3^+^ CD25^−^ CD45RO^+^ cells were seeded onto Cell-Tak–coated XFe96 cell-culture microplates with Seahorse XF RPMI media supplemented 10 mM glucose, 1 mM pyruvate, and 2 mM L-glutamine (all from Agilent Technologies). The ECAR was measured under basal conditions and upon treatment with 1.5 μM oligomycin (Oligo), 2 μM fluorocarbonyl cyanide phenylhydrazone, and 0.5 μM rotenone/antimycinA. Basal and maximum values of OCR and ECAR were calculated as described previously (Peng et al., 2016).

### T cell proliferation assay

Murine naive CD4^+^ T cells were cultured under Th1 polarized conditions in 96-well plates for 24 h, after which 18:1 LysoPS or vehicle was added to the culture; 30 h later, the cells were pulsed with 1 μCi [^3^H]-thymidine. In some experiments, 0.5 × 10^5^ or 2 × 10^5^ of in vitro–generated murine Treg cells in the presence or absence of 10 μM LysoPS were added to co-culture of 1 × 10^5^ naive CD4^+^ T cells with 1 × 10^4^ CD11c^+^ dendritic cells magnetically isolated from the spleens of C57BL/6J mice; 54 h later, the cells were pulsed with 1 μCi [^3^H]-thymidine. After 18 h, these cells were harvested onto filters, and the radioactivity was measured in a 1,450-microbeta scintillation counter (Wallac). Data are expressed in counts per min.

### ROS production assay

Intracellular ROS levels in murine in vitro–generated Th1 cells stimulated with 10 μM 18:1 LysoPS were measured by using a Cellular ROS Detection Assay Kit (Deep Red Fluorescence; Abcam). The ROS probe and 7-aminoactinomycin D were added together in PBS to the cells, and the mixture was incubated for 30 min at room temperature. Stained cells were washed with PBS, and their ROS levels were analyzed on a FACSCanto II.

### Extraction of bacterial DNA from feces

Fecal samples were collected in tubes, to which RNAlater was added to produce 10-fold dilutions of the homogenates. Fecal homogenates (200 μl) were washed twice with PBS, after which 0.3 g of glass beads (diameter, 0.1 mm), 300 μl of Tris-SDS solution, and 500 μl of Tris-EDTA–saturated phenol were added to the suspension. The resulting mixture was vortexed using a FastPrep-24 (MP Biomedicals) at power level 5.0 for 30 s. Following centrifugation of the samples at 20,000 *g* for 5 min at 4°C, phenol-chloroform extraction was performed on 400-μl aliquots of the resulting supernatants, and isopropanol precipitation was performed on 250-μl aliquots of the supernatants generated from the phenol chloroform extraction. DNA from the fecal samples was then suspended in 50 μl of Tris-EDTA buffer. Quantitative PCR was performed as described previously for the enumeration of *E. coli*/*Shigella* group in human and murine fecal samples (Pareek et al., 2019). In brief, each 20 μl reaction consisted of 5 μl of 100-fold–diluted DNA as the template and 15 μl of master mix solution containing 4.6 μl of PCR-grade water, 0.2 μl of forward and reverse primers from 10 μM stock, and 10 μl of probe GoTaq qPCR master mix (Promega). Reactions were performed with an AB Biosystems StepOnePlus System using the following program: 1 cycle of 94°C for 5 min, and 40 cycles of 94°C for 15 s, 60°C for 60 s, and 72°C for 60 s. Absolute copy numbers per gram of feces were calculated based on standard curve values obtained for *E. coli*. The following primer set was used: *E. coli*/*Shigella* group, 5′-GAG​TAA​AGT​TAA​TAC​CTT​TGC​TCA​TTG-3′ and 5′-GAGACTCAAGCTKRCCAGTATCAG-3′. Full-length *ECSF_3660* (870 bp) was detected in the microbial DNA from feces by performing PCR with 1 μl of microbial DNA from feces as the template. The following primer set was used: 5′-ATG​CGG​ACT​CTG​CAG​GGC​TGG​TTG​TTG​CCG-3′ and 5′-TCA​AAA​CAG​GTC​GTT​TAG​CAT​AAC​TCC​CAC-3′. The amplification conditions were 94°C (5 min), and 35 cycles of 94°C (30 s), 60°C (30 s), and 72°C (60 s).

### Shotgun-seq

#### Quality control (QC) of sequencing reads

We applied a series of QC steps to maximize the quality of the datasets. The main QC steps were: (i) trimming of low-quality bases, (ii) identification and masking of human reads, and (iii) removal of duplicated reads. We trimmed the raw reads to clip Illumina adapters, cut off low-quality bases at both ends, and discarded reads <60 bp in length after trimming using the Trimmomatic (version 0.33, parameters: ILLUMINACLIP:TruSeq3-PE-2.fa:2:30:10:8:true LEADING:20 TRAILING:20 MINLEN:60; Bolger et al., 2014). We aligned quality-filtered reads to the human reference genome (hg19) using bowtie2 (Langmead and Salzberg, 2012) with default parameters (version 2.3.2) and BMTagger (version 3.101; Rotmistrovsky and Agarwala, 2011). We kept only reads of which both paired ends failed to align in either tool. The average rates of host DNA contamination were 0.12% for fecal samples. As a final QC step, we removed duplicate reads using PRINSEQ-lite (v0.20.4, parameters: -derep 1; Schmieder and Edwards, 2011).

#### Taxonomic annotation of metagenome and abundance quantification

To improve both the efficiency and accuracy of taxonomic assignment, we selected the reference metagenomes of the Japanese population constructed by Nishijima et al. (2016); 6,139 genomes from the National Center for Biotechnology Information and 10 genomes from in-house complete genome data constructed at Osaka University. Furthermore, we added newly reported genomes from the cultivated human gut bacteria projects (Almeida et al., 2019; Forster et al., 2019; Zou et al., 2019). After filtration to the genomes annotated to the species with more than 50 reference genomes, the taxonomic reference genome dataset consisted of 7,881 genomes. The filtered paired-end reads were aligned to the reference genome datasets using bowtie2 with default parameters. As for multiple-mapped reads, only the best possible alignment was selected by the alignment scores. The number of reads that mapped to each genome was divided by the length of the genome. The value of each genome was summed up by each sample, and the relative abundance of each clade was calculated at six levels (L2: phylum, L3: class, L4: order, L5: family, L6: genus, L7: species). For removing batch effects indicative of contaminants, we excluded clades that had been detected in neither of our previous metagenome cohorts (31 samples with average 29 Gb per sample and 96 samples with average 8.1 Gb, respectively; Kishikawa et al., 2020). Last, we detected outlier samples by principal component analysis (PCA).

#### Functional annotation and abundance calculation

De novo assembly of the filtered paired-end reads into contigs was conducted using MEGAHIT (v1.1.2, parameters: --min-contig-len 135; Li et al., 2015). We predicted open reading frames (ORFs) on the contigs with the ab initio gene finder MetageneMark (version 3.38, parameters: -a -k -f G; Zhu et al., 2010). Next, we annotated the ORF catalog with Kyoto Encyclopedia of Genes and Genomes (KEGG; Kanehisa and Goto, 2000). For KEGG genes, we used a database of prokaryote KEGG genes and MGENES, a database of KEGG genes from metagenome samples annotated based on orthology, with a bit score >60. We aligned putative amino acid sequences translated from the ORF catalog against both databases with DIAMOND (Buchfink et al., 2015) using BLASTP (v0.9.4.105, parameters: f 6 -b 15.0–k 1 -e 1e-6 --subject-cover 50). For quantification of ORF abundance, we mapped the filtered paired-end reads to the assembled contigs using bowtie2 with default parameters. To avoid the bias of the gene size, the ORF abundance was defined as the depth of each ORF’s region of the ORF catalog according to the mapping result. As well as phylogenetic data, we excluded genes that had been detected in neither of our previous metagenome cohorts (Kishikawa et al., 2020) and detected outlier samples by PCA.

#### QC of samples

We excluded four CD samples due to high contamination rate (>90%) of host DNA, excessive duplicate reads, low mapping rate to phylogenetic reference reads, and inadequate contig formation, respectively. We further excluded two control samples due to the outlier in PCA of both phylogenetic data and gene abundance data.

#### Case–control association test for phylogenetic data

We normalized the relative abundance profiles using the Box-Cox transformation function in the car R package (v3.0.2), including log transformation. We removed clades detected (a) in <20% of the samples, (b) in no sample in either cases or controls, or (c) with an average relative abundance of <0.001% of total abundance. After selection, we assessed 801 clades (10 phyla, 23 classes, 35 orders, 73 families, 180 genera, and 480 species). Case–control association tests were performed separately for each clade using the generalized linear model function in the R package stats (v3.6.3). We adopted sex, age, and the top 15 principal components as covariates.

#### Case–control association test for gene abundance data

We converted each ORF abundance to annotated gene abundance for gene databases. We performed two steps of normalization. First, we adjusted the gene abundance by the sum of ORF abundance for each sample to correct the bias of the amount of sequence reads for each sample. Next, we applied a rank-based inverse normal transformation to correct the heterogeneity of each gene’s abundance and distribution. We removed genes detected (a) in <20% of the samples or (b) in no sample in either cases or controls. After gene selection, we assessed 162,390 genes annotated by the KEGG gene database. Case-control association tests were performed using the generalized linear model function in the R package stats (v3.6.3). We adopted sex, age, and the top 15 principal components as covariates.

#### Metagenome molecular pathway analysis

We performed gene set enrichment analysis using the R package clusterProfiler (v3.8.1). Gene sets that contained >30,000 genes or <50 genes were excluded from the enrichment analysis. For case–control pathway association tests, genes annotated by the KEGG gene database were ranked based on their effect sizes of case–control gene association tests. The KEGG gene sets were defined according to the KEGG pathway.

#### Empirical estimation of metagenome-wide significance threshold

We empirically estimated the statistical significance threshold separately for phylogenetic and gene case–control analyses, performing a phenotype permutation procedure (Kanai et al., 2016). We randomly simulated case–control phenotypes (×20,000 iterations) and calculated empirical null distributions of the minimum P values (= P_min_) in each iteration. We defined an empirical Bonferroni significance threshold, −log_10_(P_sig_), as the 95th percentile of −log_10_(P_min_) at a significance level of 0.05. We calculated −log_10_(P_sig_) using the Harrell–Davis distribution-free quantile estimator (Harrell and Davis, 1982) and calculated a 95% confidence interval for −log_10_(P_sig_) by a bootstrapping method in the R package Hmisc (v4.1.1).

#### CD case–control difference between α-diversity and β-diversity of the metagenome

For calculating diversities, all samples were down-sampled at the same number of reads (*n* = 10,000,000). α-diversity (within-sample diversity) was calculated based on gene abundance and six levels of phylogenetic relative abundance (L2–L7) for each sample according to the Shannon index. Statistical comparisons of Shannon index between CD cases and controls were assessed by Student’s *t* test. To quantify β-diversity, nonmetric multidimensional scaling on the Bray–Curtis dissimilarity was performed. For evaluating case–control differences in the dissimilarity, we performed permutational multivariate analysis of variance (PERMANOVA [McArdle and Anderson, 2001] with 100,000 permutations using the R package vegan [v2.5.4]).

### Statistical analysis

Differences between the control and experimental groups were analyzed by performing a two-tailed unpaired Student’s *t* test or one-way ANOVA followed by a Tukey’s multiple comparisons test using GraphPad Prism version 8.4.3 (GraphPad Software), except for the shotgun-seq analysis. Differences among categorical values were analyzed by performing a Fisher’s exact test using JMP software version 14.0.0 (SAS Institute). The differences for which the calculated P value was <0.05 were considered statistically significant.

### Online supplemental material

Fig. S1 shows rank-order scoring of fecal concentration of lysophospholipids in HCs and patients with CD, and ROC analysis with regard to Fig. 1 H. Fig. S2 shows effect of LysoPS injection into mice after developing 2% DSS-induced colitis on body weight loss, colon shortening, large intestinal histopathology, mRNA expression of cytokines in colonic lamina propria cells, and the number or percentage of IFN-γ^+^CD4^+^ T cells. Fig. S3 shows LysoPS-mediated promotion of mitochondria respiration in vitro–polarized murine Th1 cells; effects of LysoPS on induction of IFN-γ expression and alteration of metabolism in murine naive T cells; suppressive activities of in vitro–generated murine regulatory T cells with or without LysoPS; the impacts of mitochondrial pyruvate carrier inhibitor UK5099, the glutamine oxidation inhibitor BPTES, and fatty acid oxidation inhibitor Etomoxir on OCR in in vitro–polarized murine Th1 cells; effects of glycolytic inhibitor 2DG and UK5099 on LysoPS-induced expression of IFN-γ in human and murine Th1 cells; and the impacts of LysoPS on production of ROS and cell proliferation in murine Th1-skewed cells. Fig. S4 shows heatmaps of differentially expressed genes encoding receptor for LysoPS, TFs, and cytokines in colonic CD4^+^ T cell subsets; effects of ROCK inhibitor Fasudil on LysoPS-dependent expression of IFN-γ and HIF-1α and alterations of bioenergetic metabolism in in vitro–generated murine Th1 cells. Fig. S5 provides information about the Cas9/gRNA-targeting sites in the *P2ry10* and *P2ry10b* genes and PCR detection of wild-type or deleted loci. Table S1 lists lipid molecular species identified in feces from HCs and patients with CD. Table S2 shows fecal concentrations of lipid species upregulated in patients with CD. Table S3 lists lipid molecules determined in feces from gnotobiotic mice colonized with healthy or CD-associated microbiota. Table S4 shows concentrations of lipid species elevated in fecal samples from mice harboring microbiota from patients with CD. Table S5 lists genes with more open chromatin in LysoPS-treated Th1 cells. Table S6 lists genes with less open chromatin in in vitro–polarized Th1 cells treated with LysoPS. Table S7 shows upregulated genes in in vitro–generated Th1 cells following LysoPS treatment. Table S8 shows genes possessing more open chromatin accessibility that are transcriptionally increased in LysoPS-stimulated Th1 cells. Table S9 provides information about characteristic of the individuals participating in shotgun-seq analysis. Table S10 shows characteristics of the subjects participating in fecal lipidomic analysis and blood effector memory T cell metabolic assay. Table S11 shows internal standards used in the lipidomic analysis.

## Supplementary Material

Table S1 lists 529 lipid molecular species.

Table S2 shows concentrations of the lipid species upregulated in fecal samples from patients with CD.

Table S3 lists 121 lipid molecular species.

Table S4 shows concentrations of the lipid species upregulated in fecal samples from mice colonized with microbiota from patients with CD.

Table S5 lists genes with more open chromatin in LysoPS-treated Th1 cells.

Table S6 lists genes with less open chromatin in LysoPS-treated Th1 cells.

Table S7 shows upregulated genes in LysoPS-treated Th1 cells.

Table S8 lists genes with more open chromatin that are transcriptionally upregulated in LysoPS-stimulated Th1 cells.

Table S9 shows characteristics of the subjects participating in the shotgun-seq analysis.

Table S10 shows characteristics of patients with CD.

Table S11 lists internal standards used in the lipidomic analysis.

SourceData F2 contains original blots for Fig. 2.

SourceData FS4 contains original blots for Fig. S4.

SourceData FS5 contains original blots for Fig. S5.

## Data Availability

RNA-seq data have been deposited in the National Center Biotechnology Information Gene Expression Omnibus database (GSE169006 for Figs. 6 A, 7 E, and S4 A and Table S7). The ATAC-seq data have been deposited in DNA Data Bank of Japan database (DRA011859 for Fig. 7). The shotgun-seq data have been deposited in National Bioscience Database Center database (hum0197 for Fig. 2, A–C).
