# Supplementary material for: Lysophosphatidylserines derived from microbiota in Crohn’s disease elicit pathological Th1 response
Source: J Exp Med. 2022 May 24;219(7):e20211291. doi: 10.1084/jem.20211291 (PMC9134096; doi:10.1084/jem.20211291)
Supplement: Table S1 — lists 529 lipid molecular species. [file JEM_20211291_TableS1.docx]

**Table S1. List of 529 lipid molecular species**

|  | **Lipid class** | **Species** |
| --- | --- | --- |
| **1** | **Phosphatidylcholine**  **(PC)** | PCa32:0_(16:0/16:0) |
|  |  | PCa32:1_(16:0/16:1) |
|  |  | PCa32:2 |
|  |  | PCa34:0_(16:0/18:0) |
|  |  | PCa34:1_(16:0/18:1) |
|  |  | PCa34:2_(16:0/18:2) |
|  |  | PCa34:3_(16:0/18:3) |
|  |  | PCa35:1_(17:0/18:1) |
|  |  | PCa35:2_(17:0/18:2) |
|  |  | PCa36:0 |
|  |  | PCa36:1_(18:0/18:1) |
|  |  | PCa36:2_(18:0/18:2) |
|  |  | PCa36:3_(16:0/20:3) |
|  |  | PCa36:4_(18:2/18:2) |
|  |  | PCa36:4_(16:0/20:4) |
|  |  | PCa36:5_(16:0/20:5) |
|  |  | PCa38:0 |
|  |  | PCa38:1 |
|  |  | PCa38:2_(18:1/20:1) |
|  |  | PCa38:3_(18:0/20:3) |
|  |  | PCa38:4_(18:0/20:4) |
|  |  | PCa38:5_(16:0/22:5) |
|  |  | PCa38:6_(16:0/22:6) |
|  |  | PCa40:4 |
|  |  | PCa40:5_(18:0/22:5) |
|  |  | PCa40:6_(18:0/22:6) |
|  |  | PCa40:7 |
|  |  | PCa40:8 |
|  |  | PCa42:1 |
|  |  | PCa42:2 |
|  |  | PCa42:3 |
|  |  | PCa42:4 |
|  |  | PCa42:5 |
|  |  | PCa42:6 |
|  |  | PCa42:7 |
|  |  | PCa42:8 |
|  |  | PCa44:10 |
|  |  | PCe32:0_(O-16:0/16:0) |
|  |  | PCe34:0 |
|  |  | PCe34:1_(O-16:0/18:1) |
|  |  | PCe34:2_(O-16:0/18:2) |
|  |  | PCe34:3 |
|  |  | PCe36:1_(O-18:0/18:1) |
|  |  | PCe36:2_(O-18:1/18:1) |
|  |  | PCe38:1_(O-20:0/18:1) |
|  |  | PCe40:1_(O-22:0/18:1) |
|  |  | PCp34:1 |
|  |  | PCp34:2 |
|  |  | PCp36:1 |
|  |  | PCp36:2 |
| **2** | **Phosphatidylserine**  **(PS)** | PSa32:0_(16:0/16:0) |
|  |  | PSa32:1_(16:0/16:1) |
|  |  | PSa32:2_(16:1/16:1) |
|  |  | PSa34:0_(16:0/18:0) |
|  |  | PSa34:1_(16:0/18:1) |
|  |  | PSa34:2_(16:0/18:2) |
|  |  | PSa36:1_(18:0/18:1) |
|  |  | PSa36:2_(18:0/18:2) |
|  |  | PSa36:4_(16:0/20:4) |
|  |  | PSa37:1_(19:0/18:1) |
|  |  | PSa38:2_(18:0/20:2) |
|  |  | PSa38:3_(18:0/20:3) |
|  |  | PSa38:4_(18:0/20:4) |
|  |  | PSa38:5_(18:1/20:4) |
|  |  | PSa40:4_(18:0/22:4) |
|  |  | PSa40:5_(18:0/22:5) |
|  |  | PSa40:6_(18:0/22:6) |
| **3** | **Phosphatidylinositol**  **(PI)** | PIa34:0 |
|  |  | PIa34:1_(16:0/18:1) |
|  |  | PIa34:2_(16:0/18:2) |
|  |  | PIa34:3 |
|  |  | PIa36:0_(18:0/18:0) |
|  |  | PIa36:1_(18:0/18:1) |
|  |  | PIa36:2_(18:1/18:1) |
|  |  | PIa36:3_(18:1/18:2) |
|  |  | PIa36:4_(18:2/18:2) |
|  |  | PIa36:4_(16:0/20:4) |
|  |  | PIa36:5 |
|  |  | PIa38:2_(18:0/20:2) |
|  |  | PIa38:3_(18:0/20:3) |
|  |  | PIa38:4_(18:0/20:4) |
|  |  | PIa38:5_(18:1/20:4) |
|  |  | PIa38:6 |
|  |  | PIa38:7 |
|  |  | PIa40:4_(18:0/22:4) |
|  |  | PIa40:5_(18:0/22:5) |
| **4** | **Phosphatidylinositol-phosphate**  **(PIP)** | PIP2a38:4 |
| **5** | **Phosphatidic acid**  **(PA)** | PAa32:0_(16:0/16:0) |
|  |  | PAa32:1_(16:0/16:1) |
|  |  | PAa32:2 |
|  |  | PAa34:1_(16:0/18:1) |
|  |  | PAa34:2_(16:0/18:2) |
|  |  | PAa34:3 |
|  |  | PAa36:1_(18:0/18:1) |
|  |  | PAa36:2_(18:1/18:1) |
|  |  | PAa36:3_(18:1/18:2) |
|  |  | PAa36:4_(18:2/18:2) |
|  |  | PAa36:4_(16:0/20:4) |
|  |  | PAa38:1_(18:0/20:1) |
|  |  | PAa38:3_(18:0/20:3) |
|  |  | PAa38:4_(18:0/20:4) |
|  |  | PAa38:5_(18:1/20:4) |
|  |  | PAa38:6_(16:0/22:6) |
|  |  | PAa40:4_(18:0/22:4) |
|  |  | PAa40:5_(20:1/20:4) |
|  |  | PAa40:6 |
|  |  | PAa40:7 |
|  |  | PAa40:8 |
|  |  | PAa42:6 |
|  |  | PAa42:7 |
|  |  | PAa42:8 |
| **6** | **Lysophosphatidylcholine**  **(LysoPC)** | LPCa14:0 |
|  |  | LPCa16:0 |
|  |  | LPCa16:1 |
|  |  | LPCa18:0 |
|  |  | LPCa18:0 |
|  |  | LPCa18:1 |
|  |  | LPCa18:2 |
|  |  | LPCa18:3 |
|  |  | LPCa20:0 |
|  |  | LPCa20:1 |
|  |  | LPCa20:2 |
|  |  | LPCa20:3 |
|  |  | LPCa20:4 |
|  |  | LPCa20:5 |
|  |  | LPCa22:0 |
|  |  | LPCa22:1 |
|  |  | LPCa22:4 |
|  |  | LPCa22:5 |
|  |  | LPCa22:6 |
|  |  | LPCa24:0 |
|  |  | LPCa24:1 |
|  |  | LPCe16:0 |
|  |  | LPCe18:0 |
|  |  | LPCe18:1 |
|  |  | LPCe20:0 |
|  |  | LPCe20:1 |
|  |  | LPCe20:2 |
|  |  | LPCe22:1 |
|  |  | LPCe22:2 |
|  |  | LPCp16:0 |
|  |  | LPCp18:0 |
|  |  | LPCp18:1 |
|  |  | LPCp20:0 |
|  |  | LPCp20:1 |
|  |  | LPCp22:1 |
| **7** | **Phosphatidylserine**  **(LysoPS)** | LPSa14:0 |
|  |  | LPSa16:0 |
|  |  | LPSa16:1 |
|  |  | LPSa18:0 |
|  |  | LPSa18:1 |
|  |  | LPSa18:2 |
|  |  | LPSa18:3 |
|  |  | LPSa19:0 |
|  |  | LPSa20:0 |
|  |  | LPSa20:1 |
|  |  | LPSa20:2 |
|  |  | LPSa20:3 |
|  |  | LPSa20:4 |
|  |  | LPSa21:0 |
|  |  | LPSa22:0 |
|  |  | LPSa22:1 |
|  |  | LPSa22:2 |
|  |  | LPSa22:6 |
| **8** | **Lysophosphatidylinositol**  **(LysoPI)** | LPIa14:0 |
|  |  | LPIa16:0 |
|  |  | LPIa18:0 |
|  |  | LPIa18:1 |
|  |  | LPIa18:2 |
|  |  | LPIa18:3 |
|  |  | LPIa20:0 |
|  |  | LPIa20:1 |
|  |  | LPIa20:4 |
|  |  | LPIa20:5 |
| **9** | **Lysophosphatidylglycerol**  **(LysoPG)** | LPGa14:0 |
|  |  | LPGa16:0 |
|  |  | LPGa16:1 |
|  |  | LPGa18:0 |
|  |  | LPGa18:1 |
|  |  | LPGa18:2 |
|  |  | LPGa18:3 |
|  |  | LPGa20:0 |
|  |  | LPGa20:1 |
|  |  | LPGa20:2 |
|  |  | LPGa20:3 |
|  |  | LPGa20:4 |
|  |  | LPGa22:0 |
|  |  | LPGa22:1 |
|  |  | LPGa22:5 |
|  |  | LPGa22:6 |
| **10** | **Lysophosphatidic acid**  **(LysoPA)** | LPAa14:0 |
|  |  | LPAa16:0 |
|  |  | LPAa16:1 |
|  |  | LPAa18:0 |
|  |  | LPAa18:1 |
|  |  | LPAa18:2 |
|  |  | LPAa18:3 |
|  |  | LPAa19:0 |
|  |  | LPAa20:0 |
|  |  | LPAa20:1 |
|  |  | LPAa20:2 |
|  |  | LPAa20:3 |
|  |  | LPAa20:4 |
|  |  | LPAa20:5 |
|  |  | LPAa21:0 |
|  |  | LPAa22:0 |
|  |  | LPAa22:1 |
|  |  | LPAa22:5 |
|  |  | LPAa22:6 |
|  |  | LPAa24:0 |
|  |  | LPAa24:1 |
|  |  | LPAa24:5 |
|  |  | LPAa24:6 |
| **11** | **Fatty acid**  **(FA)** | FA6:0 |
|  |  | FA8:0 |
|  |  | FA10:0 |
|  |  | FA12:0 |
|  |  | FA14:0 |
|  |  | FA16:0 |
|  |  | FA16:1 |
|  |  | FA18:0 |
|  |  | FA18:1 |
|  |  | FA18:2 |
|  |  | FA18:3 |
|  |  | FA20:0 |
|  |  | FA20:1 |
|  |  | FA20:2 |
|  |  | FA20:3 |
|  |  | FA20:3 |
|  |  | FA20:4 |
|  |  | FA20:5 |
|  |  | FA22:0 |
|  |  | FA22:1 |
|  |  | FA22:2 |
|  |  | FA22:3 |
|  |  | FA22:4 |
|  |  | FA22:5 |
|  |  | FA22:6 |
|  |  | FA24:0 |
|  |  | FA24:1 |
|  |  | FA24:5 |
|  |  | FA24:6 |
|  |  | FA26:0 |
| **12** | **Sphingomyeline**  **(SM)** | SM_d18:1/16:0 |
|  |  | SM_d18:1/16:1 |
|  |  | SM_d18:1/18:0 |
|  |  | SM_d18:1/18:1 |
|  |  | SM_d18:1/19:0 |
|  |  | SM_d18:1/20:0 |
|  |  | SM_d18:1/20:1 |
|  |  | SM_d18:1/21:0 |
|  |  | SM_d18:1/21:1 |
|  |  | SM_d18:1/22:0 |
|  |  | SM_d18:1/22:1 |
|  |  | SM_d18:1/22:2 |
|  |  | SM_d18:1/23:0 |
|  |  | SM_d18:1/23:1 |
|  |  | SM_d18:1/24:0 |
|  |  | SM_d18:1/24:1 |
|  |  | SM_d18:1/24:2 |
|  |  | SM_d18:1/25:0 |
|  |  | SM_d18:1/26:0 |
|  |  | SM_d18:1/26:1 |
|  |  | SM_d18:1/26:2 |
|  |  | SM_d18:0/16:0 |
|  |  | SM_d18:0/18:0 |
|  |  | SM_d18:0/20:0 |
|  |  | SM_d18:0/22:0 |
|  |  | SM_d18:0/24:0 |
|  |  | SM_d18:0/24:1 |
| **13** | **Ceramide**  **(Cer)** | Cer_d18:1/16:0 |
|  |  | Cer_d18:1/16:1 |
|  |  | Cer_d18:1/18:0 |
|  |  | Cer_d18:1/18:1 |
|  |  | Cer_d18:1/18:2 |
|  |  | Cer_d18:1/20:0 |
|  |  | Cer_d18:1/20:1 |
|  |  | Cer_d18:1/22:0 |
|  |  | Cer_d18:1/22:1 |
|  |  | Cer_d18:1/22:2 |
|  |  | Cer_d18:1/23:0 |
|  |  | Cer_d18:1/24:0 |
|  |  | Cer_d18:1/24:1 |
|  |  | Cer_d18:2/24:1 |
|  |  | Cer_d18:1/25:0 |
|  |  | Cer_d18:1/26:0 |
|  |  | Cer_d18:0/16:0 |
|  |  | Cer_d18:0/18:0 |
|  |  | Cer_d18:0/20:0 |
|  |  | Cer_d18:0/22:0 |
|  |  | Cer_d18:0/24:0 |
|  |  | Cer_d18:0/24:1 |
|  |  | Cer_d18:0/26:0 |
| **14** | **Hydroxyceramide**  **Cer(OH)** | Cer(OH)(d42:1) |
| **15** | **Hexosylceramide**  **(HexCer)** | HexCer_d18:1/16:0 |
|  |  | HexCer_d18:1/18:0 |
|  |  | HexCer_d18:1/18:1 |
|  |  | HexCer_d18:1/20:0 |
|  |  | HexCer_d18:1/21:0 |
|  |  | HexCer_d18:1/22:0 |
|  |  | HexCer_d18:1/22:1 |
|  |  | HexCer_d18:1/22:2 |
|  |  | HexCer_d18:1/23:0 |
|  |  | HexCer_d18:1/24:0 |
|  |  | HexCer_d18:1/24:1 |
|  |  | HexCer_d18:1/24:2 |
|  |  | HexCer_d18:1/26:0 |
|  |  | HexCer_d18:0/16:0 |
|  |  | HexCer_d18:0/18:0 |
|  |  | HexCer_d18:0/20:0 |
|  |  | HexCer_d18:0/22:0 |
|  |  | HexCer_d18:0/24:0 |
|  |  | HexCer_d18:0/24:1 |
|  |  | HexCer_d18:0/26:0 |
| **16** | **Hexosyl(hydroxy)ceramide**  **(HexCer(OH))** | HexCer(OH)(d36:2) |
|  |  | HexCer(OH)(d40:1) |
|  |  | HexCer(OH)(d42:1) |
|  |  | HexCer(OH)(d42:2) |
|  |  | HexCer(OH)(d42:3) |
| **17** | **Dihexosylceramide**  **(Hex2Cer)** | Hex2Cer_d18:1/18:0 |
|  |  | Hex2Cer_d18:1/20:0 |
|  |  | Hex2Cer_d18:1/22:0 |
|  |  | Hex2Cer_d18:1/22:1 |
|  |  | Hex2Cer_d18:1/22:2 |
|  |  | Hex2Cer_d18:1/23:0 |
|  |  | Hex2Cer_d18:1/24:0 |
|  |  | Hex2Cer_d18:1/24:1 |
|  |  | Hex2Cer_d18:1/24:2 |
|  |  | Hex2Cer_d18:1/26:0 |
| **18** | **Salfatide**  **(ST)** | Sulfatide_d18:1/14:0 |
|  |  | Sulfatide_d18:1/16:0 |
|  |  | Sulfatide_d18:1/16:1 |
|  |  | Sulfatide_d18:1/18:0 |
|  |  | Sulfatide_d18:1/18:1 |
|  |  | Sulfatide_d18:1/20:0 |
|  |  | Sulfatide_d18:1/20:1 |
|  |  | Sulfatide_d18:1/22:1 |
|  |  | Sulfatide_d18:1/24:2 |
|  |  | Sulfatide_d18:1/26:0 |
| **19** | **Dihydrosalfatide**  **(ST(OH))** | Sulfatide(OH)(d34:1) |
|  |  | Sulfatide(OH)(d34:2) |
|  |  | Sulfatide(OH)(d36:1) |
|  |  | Sulfatide(OH)(d36:2) |
|  |  | Sulfatide(OH)(d38:1) |
|  |  | Sulfatide(OH)(d38:2) |
|  |  | Sulfatide(OH)(d40:1) |
|  |  | Sulfatide(OH)(d40:2) |
|  |  | Sulfatide(OH)(d42:1) |
|  |  | Sulfatide(OH)(d42:2) |
|  |  | Sulfatide(OH)(d42:3) |
|  |  | Sulfatide(OH)(d44:1) |
| **20** | **Phosphatidylglycerol**  **(PG)** | PGa32:0_(16:0/16:0) |
|  |  | PGa32:1_(16:0/16:1) |
|  |  | PGa33:0 |
|  |  | PGa33:1 |
|  |  | PGa34:0_(16:0/18:0) |
|  |  | PGa34:1_(16:0/18:1) |
|  |  | PGa34:2_(16:0/18:2) |
|  |  | PGa34:3 |
|  |  | PGa36:0 |
|  |  | PGa36:1_(18:0/18:1) |
|  |  | PGa36:2_(18:1/18:1) |
|  |  | PGa36:3_(18:1/18:2) |
|  |  | PGa36:4_(16:0/20:4) |
|  |  | PGa38:1 |
|  |  | PGa38:2 |
|  |  | PGa38:3 |
|  |  | PGa38:4_(18:0/20:4) |
|  |  | PGa38:5 |
|  |  | PGa40:4_(18:0/22:4) |
|  |  | PGa40:6_(18:0/22:6) |
| **21** | **Diacylglycerol**  **(DAG)** | DAG34:0 |
|  |  | DAG34:0 |
|  |  | DAG34:1_(16:0/18:1)sn-1,3 |
|  |  | DAG34:1_(16:0/18:1)sn-1,2 |
|  |  | DAG34:2_(16:0/18:2)sn-1,3 |
|  |  | DAG34:2_(16:0/18:2)sn-1,2 |
|  |  | DAG34:3_(16:0/18:3) |
|  |  | DAG34:3 |
|  |  | DAG34:3 |
|  |  | DAG36:1 |
|  |  | DAG36:1 |
|  |  | DAG36:2_(18:1/18:1)sn-1,3 |
|  |  | DAG36:2_(18:1/18:1)sn-1,2 |
|  |  | DAG36:3_(18:1/18:2)sn-1,3 |
|  |  | DAG36:3_(18:1/18:2)sn-1,2 |
|  |  | DAG36:3 |
|  |  | DAG36:4_(18:2/18:2)sn-1,3 |
|  |  | DAG36:4_(18:2/18:2)sn-1,2 |
|  |  | DAG38:0 |
|  |  | DAG38:1 |
|  |  | DAG38:2 |
|  |  | DAG38:2 |
|  |  | DAG38:3 |
|  |  | DAG38:3 |
|  |  | DAG38:4 |
|  |  | DAG38:4 |
|  |  | DAG38:5 |
|  |  | DAG38:5 |
|  |  | DAG38:6_(16:0/22:6) |
|  |  | DAG38:6_(16:0/22:6) |
|  |  | DAG38:7 |
|  |  | DAG38:7 |
|  |  | DAG40:1 |
|  |  | DAG40:2 |
|  |  | DAG40:3 |
|  |  | DAG40:4 |
| **22** | **Monoacylglycerol**  **(MAG)** | MAG16:0_sn-1 |
|  |  | MAG16:1 |
|  |  | MAG18:0_sn-1 |
|  |  | MAG18:1_sn-2 |
|  |  | MAG18:1_sn-1 |
|  |  | MAG18:2_sn-2 |
|  |  | MAG18:2_sn-1 |
|  |  | MAG18:3 |
|  |  | MAG20:2 |
|  |  | MAG20:3 |
|  |  | MAG20:4_sn-2 |
| **23** | **N-Acylethanolamine**  **(AEA)** | AEA14:0 |
|  |  | AEA15:0 |
|  |  | AEA16:0 |
|  |  | AEA16:1 |
|  |  | AEA17:0 |
|  |  | AEA18:0 |
|  |  | AEA18:1 |
|  |  | AEA18:2 |
|  |  | AEA18:3 |
|  |  | AEA19:0 |
|  |  | AEA20:0 |
|  |  | AEA20:1 |
|  |  | AEA20:2 |
|  |  | AEA20:3 |
|  |  | AEA22:0 |
|  |  | AEA22:4 |
|  |  | AEA24:0 |
|  |  | AEA24:1 |
|  |  | AEA25:0 |
| **24** | **Acylcarnitine**  **(AC)** | ACC2:0 |
|  |  | ACC3:0COOH |
|  |  | ACC3:0 |
|  |  | ACC4:0 |
|  |  | ACC5:0 |
|  |  | ACC6:0 |
|  |  | ACC7:0 |
|  |  | ACC8:0 |
|  |  | ACC9:0 |
|  |  | ACC10:0 |
|  |  | ACC12:0 |
|  |  | ACC13:0 |
|  |  | ACC14:0 |
|  |  | ACC16:0 |
|  |  | ACC16:1 |
|  |  | ACC18:0 |
|  |  | ACC18:1 |
|  |  | ACC18:2 |
|  |  | ACC20:0 |
|  |  | ACC22:6 |
| **25** | **Cholesterol**  **(CHO)** | Cholesterol |
| **26** | **Cholesterolester**  **(CE)** | CEa18:2 |
|  |  | CEa20:4 |
| **27** | **Phosphatidylethanolamine**  **(PEa)** | PEa32:0 |
|  |  | PEa32:0_(16:0/16:0) |
|  |  | PEa34:1_(16:0/18:1) |
|  |  | PEa34:2 |
|  |  | PEa34:4 |
|  |  | PEa36:1_(18:0/18:1) |
|  |  | PEa36:2_(18:0/18:2) |
|  |  | PEa36:3_(18:1/18:2) |
|  |  | PEa38:1_(18:0/20:1) |
|  |  | PEa38:2_(18:1/20:1) |
|  |  | PEa38:3 |
|  |  | PEa38:4_(16:0/22:4) |
|  |  | PEa38:4_(18:0/20:4) |
|  |  | PEa38:5_(18:1/20:4) |
|  |  | PEa40:1 |
|  |  | PEa40:2 |
|  |  | PEa40:3 |
|  |  | PEa40:4_(18:0/22:4) |
|  |  | PEa40:5_(18:1/22:4) |
|  |  | PEa40:5_(18:0/22:5) |
|  |  | PEa40:6_(18:0/22:6) |
|  |  | PEa42:2 |
|  |  | PEa42:3 |
|  |  | PEa42:4 |
|  |  | PEa42:6 |
|  |  | PEa42:7 |
|  |  | PEp32:1_(P-16:0/16:1) |
|  |  | PEp34:1_(P-16:0/18:1) |
|  |  | PEp34:2_(P-16:0/18:2) |
|  |  | PEp38:4_(P-18:0/20:4) |
|  |  | PEp40:1_(P-22:0/18:1) |
|  |  | PEp40:2_(P-22:1/18:1) |
|  |  | PEp40:3_(P-22:1/18:2) |
|  |  | PEp40:4_(P-18:0/22:4) |
|  |  | PEp40:5_(P-18:0/22:5) |
| **28** | **Lysophosphatidylethanolamine**  **(LPE)** | LPEa16:0 |
|  |  | LPEa16:1 |
|  |  | LPEa18:0 |
|  |  | LPEa18:1 |
|  |  | LPEa18:2 |
|  |  | LPEa18:3 |
|  |  | LPEa20:0 |
|  |  | LPEa22:0 |
|  |  | LPEa24:0 |
|  |  | LPEa24:1 |
| **29** | **Bis(monoacylglycero)phosphate**  **(BMP)** | BMPa(16:0/16:0) |
|  |  | BMPa(16:0/18:0) |
|  |  | BMPa(16:0/18:1) |
|  |  | BMPa(18:0/18:0) |
|  |  | BMPa(18:0/18:1) |
|  |  | BMPa(18:1/18:1) |
|  |  | BMPa(18:1/18:2) |
|  |  | BMPa(20:4/22:6) |
|  |  | BMPa(22:6/22:6) |
| **30** | **Acyl Coenzyme A**  **(AcylCoA)** | C8:0-CoA |
|  |  | C10:0-CoA |
|  |  | C18:1-CoA |
|  |  | C20:3-CoA |
|  |  | C20:4-CoA |
|  |  | C20:5-CoA |
|  |  | C22:5-CoA |
| **31** | **Sphingosine-1-phosphate**  **(S1P)** | Sph(d18:1/0:0)P_S1P |
|  |  | Sph(d18:0/0:0)P_So1P |
| **32** | **Sphingosine**  **(Sph)** | Sph(d18:2/0:0)_SPD |
|  |  | Sph(d18:1/0:0)_Sphingosine |
|  |  | Sph(d18:0/0:0)_Sphinganine |
|  |  | Sph(d20:1/0:0) |
| **33** | **Hexosylsphingosine**  **(HexSph)** | HexSph(d18:1/0:0) |
| **34** | **Lactosylsphingosine**  **(LacSph)** | LacSph(d18:1/0:0) |

a, acyl-linked; p, plasmalogen-linked; e, alkyl ether-linked.
