# Supplementary material for: Lysophosphatidylserines derived from microbiota in Crohn’s disease elicit pathological Th1 response
Source: J Exp Med. 2022 May 24;219(7):e20211291. doi: 10.1084/jem.20211291 (PMC9134096; doi:10.1084/jem.20211291)
Supplement: Table S3 — lists 121 lipid molecular species. [file JEM_20211291_TableS3.docx]

**Table S3. List of 121 lipid molecular species**

| 1 | PSa | PSa32:2 |
| --- | --- | --- |
|  |  | PSa34:1 |
|  |  | PSa34:2 |
|  |  | PSa34:3 |
|  |  | PSa36:1 |
|  |  | PSa36:2 |
|  |  | PSa36:3 |
|  |  | PSa36:4 |
|  |  | PSa36:5 |
|  |  | PSa38:2 |
|  |  | PSa38:3 |
|  |  | PSa38:4 |
|  |  | PSa38:5 |
|  |  | PSa38:6 |
|  |  | PSa40:2 |
|  |  | PSa40:3 |
|  |  | PSa40:4 |
|  |  | PSa40:5 |
|  |  | PSa40:6 |
|  |  | PSa40:8 |
|  |  | PSa42:10 |
| 2 | LPC | LPCa16:0 |
|  |  | LPCa16:1 |
|  |  | LPCa18:0 |
|  |  | LPCa18:1 |
|  |  | LPCa18:2 |
|  |  | LPCa20:0 |
|  |  | LPCa20:1 |
|  |  | LPCa20:2 |
|  |  | LPCa20:3 |
|  |  | LPCa20:4 |
|  |  | LPCa20:5 |
|  |  | LPCa22:0 |
|  |  | LPCa22:1 |
|  |  | LPCa22:2 |
|  |  | LPCa22:3 |
|  |  | LPCa22:4 |
|  |  | LPCa22:5 |
|  |  | LPCa22:6 |
|  |  | LPCa24:0 |
|  |  | LPCa24:1 |
|  |  | LPCa24:2 |
|  |  | LPCa24:3 |
|  |  | LPCa24:4 |
|  |  | LPCa24:5 |
|  |  | LPCa24:6 |
| 3 | LPS | LPSa16:0 |
|  |  | LPSa18:0 |
|  |  | LPSa18:1 |
|  |  | LPSa18:2 |
|  |  | LPSa20:0 |
|  |  | LPSa20:4 |
|  |  | LPSa22:0 |
|  |  | LPSa22:1 |
|  |  | LPSa22:2 |
|  |  | LPSa22:5 |
|  |  | LPSa22:6 |
| 4 | LPI | LPIa16:0 |
|  |  | LPIa18:0 |
|  |  | LPIa18:1 |
|  |  | LPIa18:2 |
|  |  | LPIa20:4 |
|  |  | LPIa22:6 |
| 5 | LPG | LPGa16:0 |
|  |  | LPGa16:1 |
|  |  | LPGa18:0 |
|  |  | LPGa18:1 |
|  |  | LPGa18:2 |
|  |  | LPGa20:0 |
|  |  | LPGa20:1 |
|  |  | LPGa20:2 |
|  |  | LPGa20:3 |
|  |  | LPGa20:4 |
|  |  | LPGa20:5 |
|  |  | LPGa22:0 |
|  |  | LPGa22:1 |
|  |  | LPGa22:6 |
| 6 | LPA | LPAa14:0 |
|  |  | LPAa16:0 |
|  |  | LPAa16:1 |
|  |  | LPAa18:0 |
|  |  | LPAa18:1 |
|  |  | LPAa18:2 |
|  |  | LPAa18:3 |
|  |  | LPAa20:0 |
|  |  | LPAa20:1 |
|  |  | LPAa20:2 |
|  |  | LPAa20:4 |
|  |  | LPAa22:0 |
|  |  | LPAa22:1 |
|  |  | LPAa22:5 |
|  |  | LPAa22:6 |
| 7 | FA | FA14:0 |
|  |  | FA14:1 |
|  |  | FA14:2 |
|  |  | FA16:0 |
|  |  | FA16:1 |
|  |  | FA16:2 |
|  |  | FA18:0 |
|  |  | FA18:1_OA |
|  |  | FA18:1_VA |
|  |  | FA18:2 |
|  |  | FA18:3 |
|  |  | FA20:0 |
|  |  | FA20:1 |
|  |  | FA20:2 |
|  |  | FA20:3_n3 |
|  |  | FA20:3_n6 |
|  |  | FA20:4_n3 |
|  |  | FA20:4_n6 |
|  |  | FA20:5_n3 |
|  |  | FA22:0 |
|  |  | FA22:1 |
|  |  | FA22:4 |
|  |  | FA22:5 |
|  |  | FA22:6 |
|  |  | FA24:0 |
|  |  | FA24:1 |
|  |  | FA24:5 |
|  |  | FA24:6 |
|  |  | FA26:0 |

PSa, acyl-linked phosphatidylserine; LPC, lysophosphatidylcholine; LPS, lysophosphatidylserine; LPI, lysophosphatidylinositol; LPG, lysophosphatidylglycerol; LPA, lysophosphatidic acid; FA, fatty acid.
