# Supplementary material for: Lysophosphatidylserines derived from microbiota in Crohn’s disease elicit pathological Th1 response
Source: J Exp Med. 2022 May 24;219(7):e20211291. doi: 10.1084/jem.20211291 (PMC9134096; doi:10.1084/jem.20211291)
Supplement: Table S8 — lists genes with more open chromatin that are transcriptionally upregulated in LysoPS-stimulated Th1 cells. [file JEM_20211291_TableS8.docx]

**Table S8. List of the genes with more open chromatin that are transcriptionally upregulated in LysoPS-stimulated Th1 cells**

| **Gzma** | **Il17f** | **Nqo2** | **Lrrc8e** |
| --- | --- | --- | --- |
| **Muc13** | **Itga7** | **Bcl2l15** | **Ctla2b** |
| **Serpinb5** | **Tmem163** | **Alpk2** | **Nfia** |
| **Pparg** | **Car5b** | **Lilr4b** | **Nol4l** |
| **Upp1** | **Tns4** | **Abcc3** | **Ecm1** |
| **Cdh17** | **Il1r2** | **Ccdc74a** | **Il24** |
| **Slc44a3** | **Mt3** | **Tlr5** | **Serpinf1** |
| **Pde1a** | **Cyp11a1** | **Kcnab3** | **Gcnt1** |
| **Myo1f** | **Il1r1** | **Fbxo15** | **Fut4** |
| **Insl5** | **Zmynd10** | **S100a6** | **B3gnt8** |
| **Klrc1** | **H2-Q2** | **Lilrb4a** | **Slc10a1** |
| **Pigz** | **Clic5** | **Ankrd34b** | **Ffar2** |
| **Klrb1b** | **Pdpn** | **Kcnj8** | **Casp6** |
| **Rorc** | **Prf1** | **Catsper2** | **M1ap** |
| **Ltb4r1** | **Cxcr2** | **Nradd** | **Ppt1** |
| **Ifitm5** | **Spats2l** | **Ociad2** | **Glrx** |
| **Lair1** | **Tgfb3** | **Tgm1** | **S100a3** |
| **Slc38a3** | **S100a4** | **4930486L24Rik** | **Tnfrsf8** |
| **Bst1** | **Adora3** | **Ctla2a** | **Spo11** |
| **Gm5861** | **Prdm1** | **Egln3** | **Tgm2** |
| **Casr** | **Icosl** | **Cldn10** | **Pde2a** |
| **Emp1** | **Myo1h** | **Endou** | **B430306N03Rik** |
| **Tmem40** | **Il21** | **Hip1** |  |
| **Il23r** | **P2ry14** | **Atp6v0d2** |  |
| **1700056E22Rik** | **Itgam** | **Plac8** |  |
