# Supplementary figures and images for: Lysophosphatidylserines derived from microbiota in Crohn’s disease elicit pathological Th1 response
Source: J Exp Med. 2022 May 24;219(7):e20211291. doi: 10.1084/jem.20211291 (PMC9134096; doi:10.1084/jem.20211291)

F

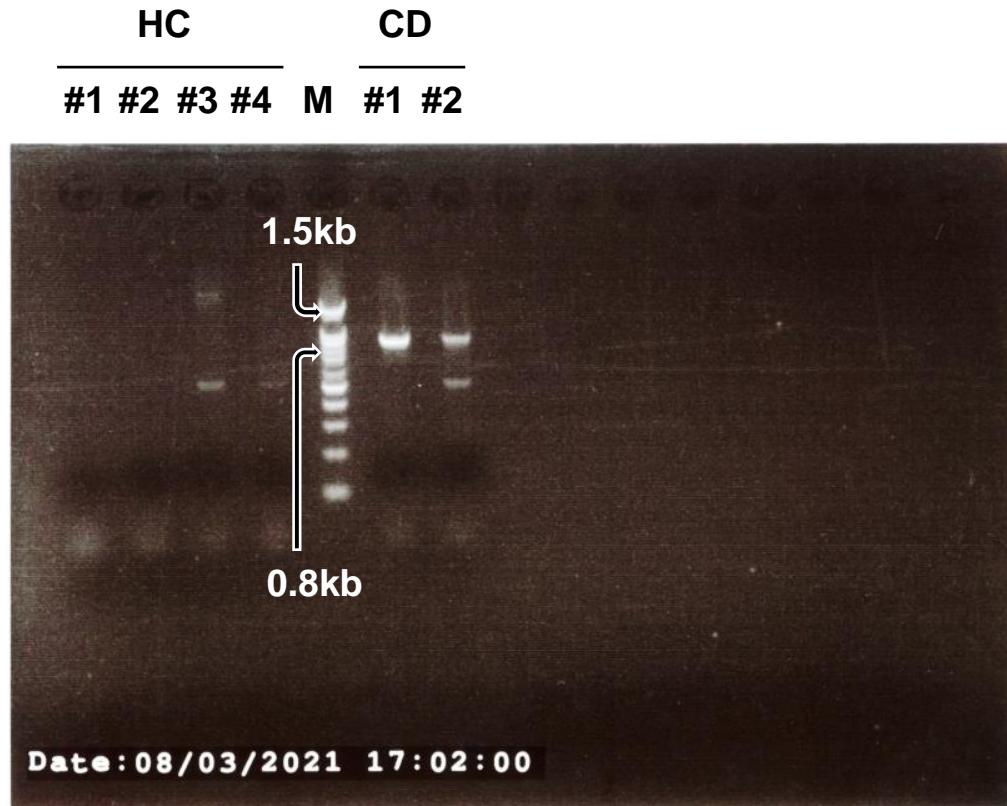

H

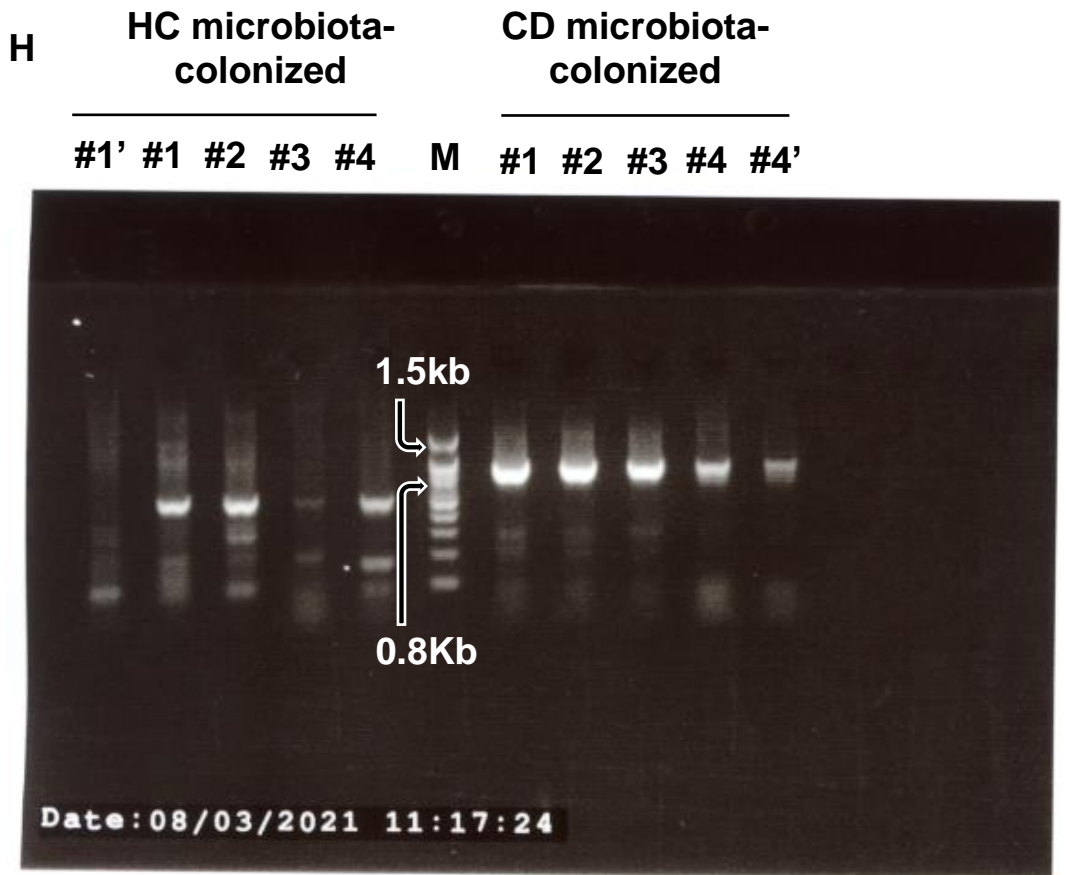

Supplement: SourceData F2 — contains original blots for Fig. 2. [file JEM_20211291_SourceDataF2.pdf]

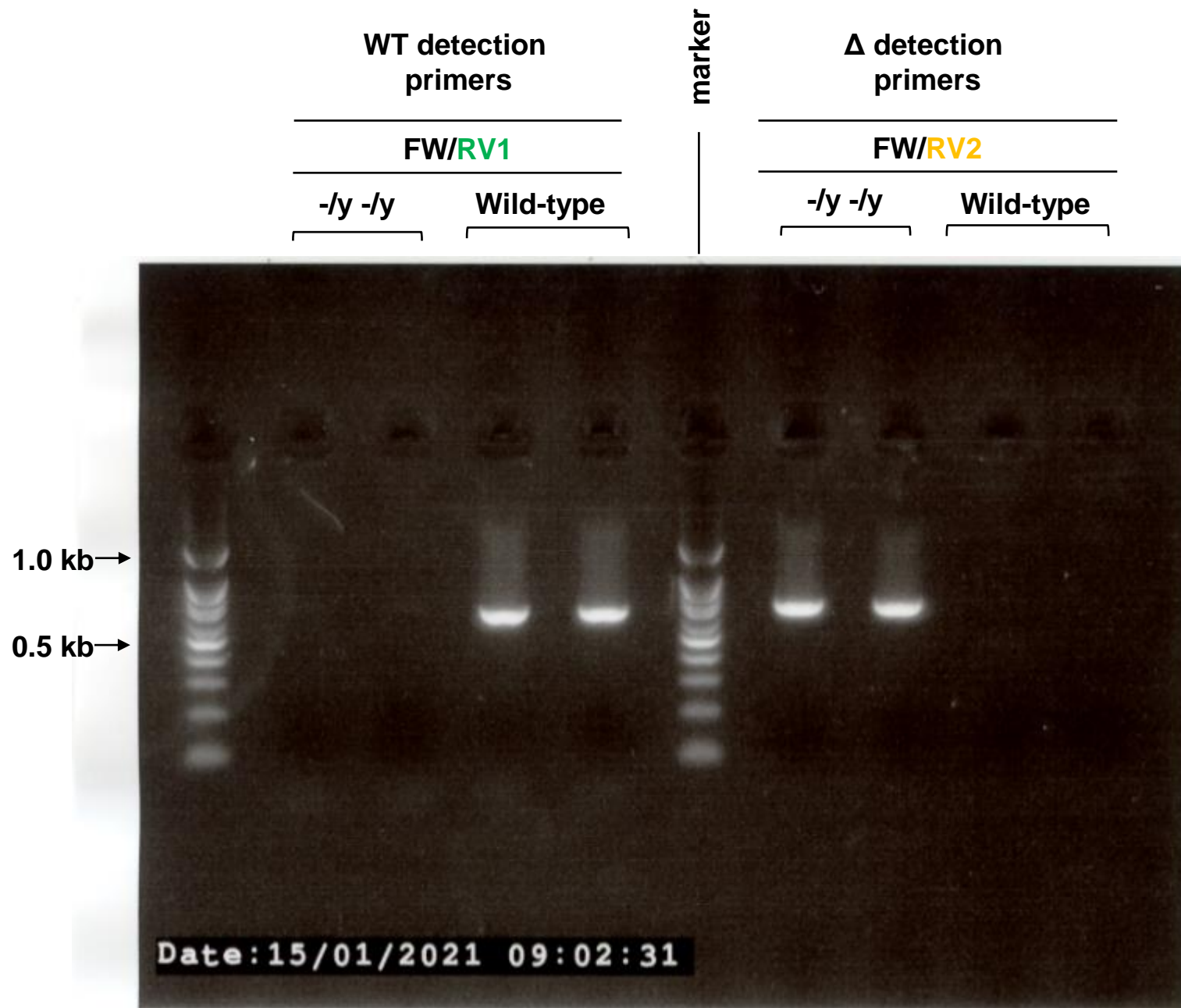

Supplement: SourceData FS5 — contains original blots for Fig. S5. [file JEM_20211291_SourceDataFS5.pdf]
