# Supplementary material for: Lysophosphatidylserines derived from microbiota in Crohn’s disease elicit pathological Th1 response
Source: J Exp Med. 2022 May 24;219(7):e20211291. doi: 10.1084/jem.20211291 (PMC9134096; doi:10.1084/jem.20211291)
Supplement: SourceData FS4 — contains original blots for Fig. S4. [file JEM_20211291_SourceDataFS4.pdf]

HIF-1α

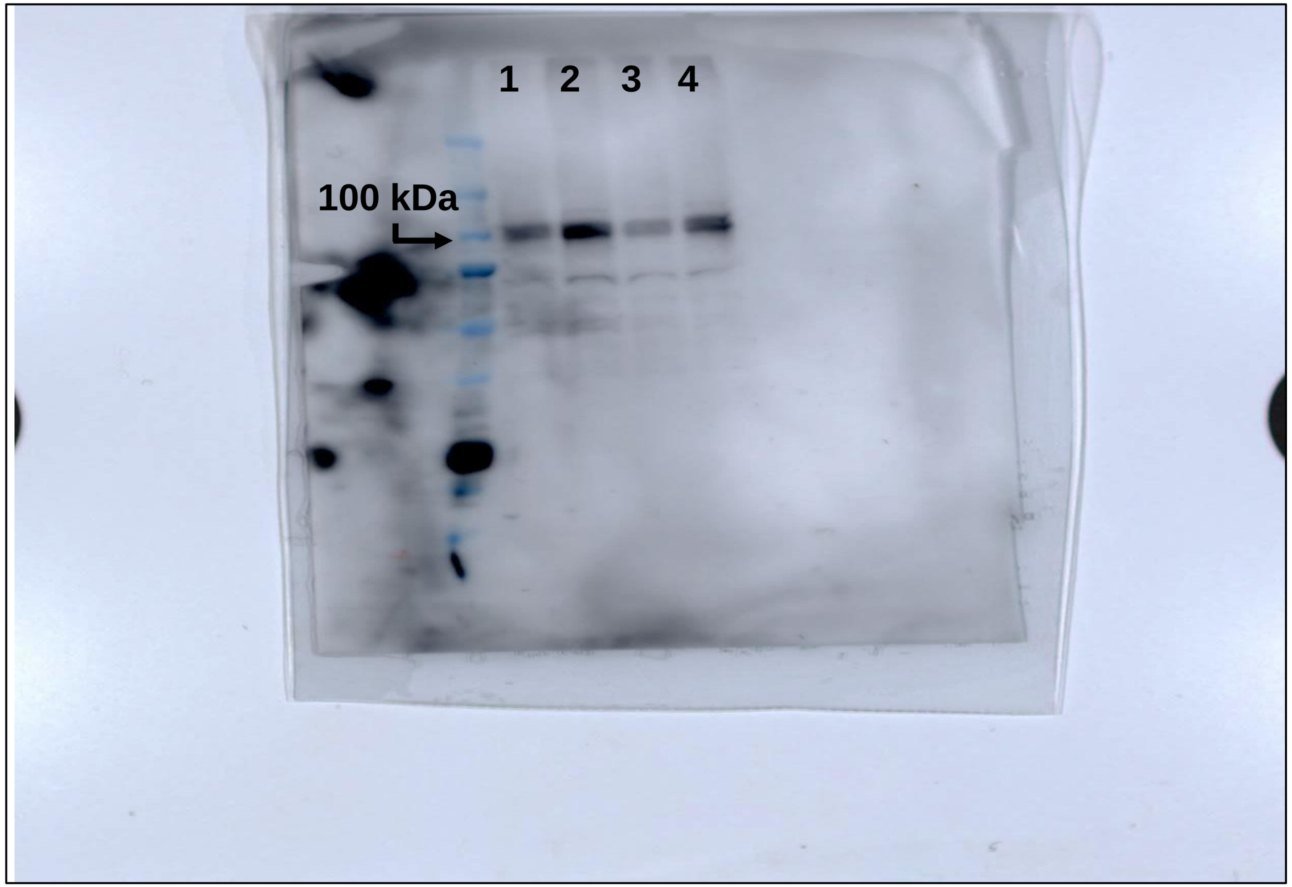

β-actine

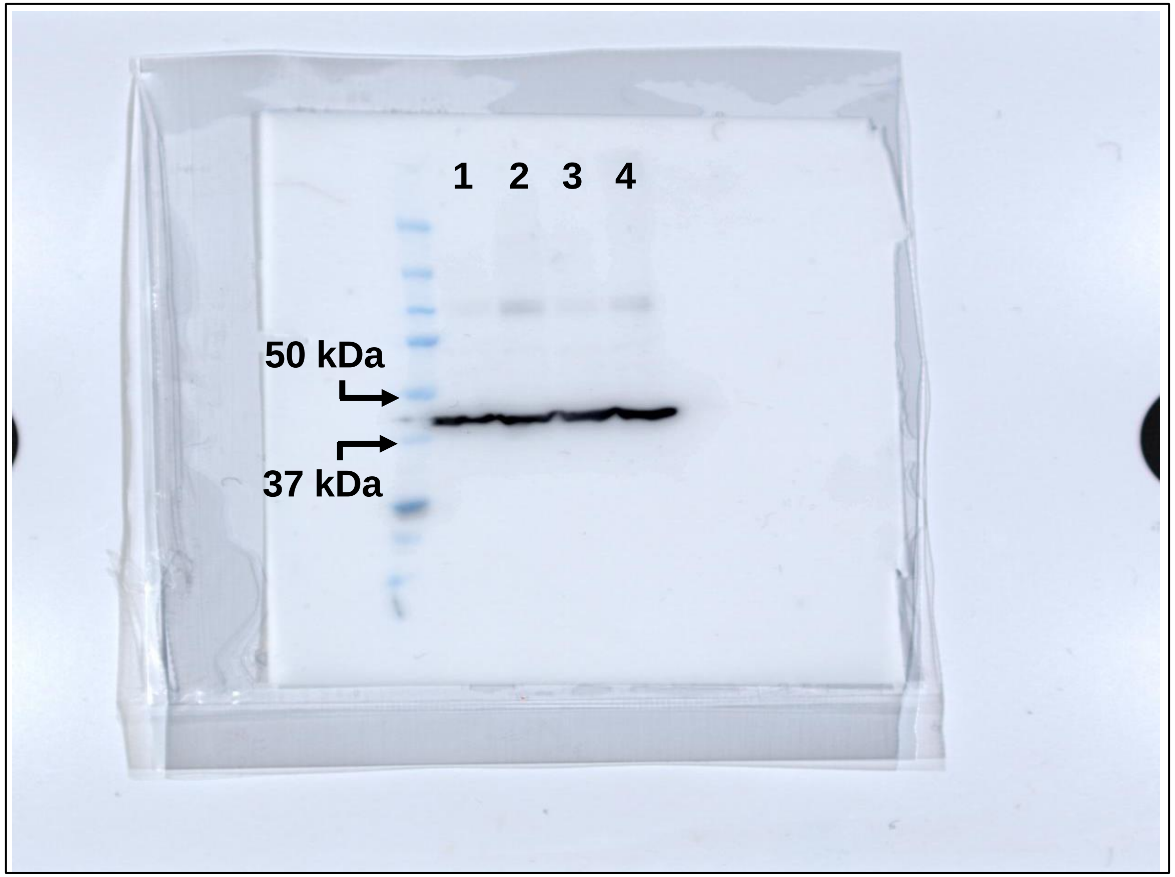

Lane 1: Fasudil (-), LysoPS (-)
Lane 2: Fasudil (-), LysoPS (+)
Lane 3:Fasudil (+), LysoPS (-)
Lane 4: Fasudil (+), LysoPS (+)

Lane 1: Fasudil (-), LysoPS (-)
Lane 2: Fasudil (-), LysoPS (+)
Lane 3:Fasudil (+), LysoPS (-)
Lane 4: Fasudil (+), LysoPS (+)
